# Safety and immunogenicity of a killed bivalent (O1 and O139) whole-cell oral cholera vaccine in adults and children in Vellore, South India

**DOI:** 10.1371/journal.pone.0218033

**Published:** 2019-06-18

**Authors:** Venkata Raghava Mohan, Santosh Raj, Mandeep Singh Dhingra, Naveena Aloysia D’Cor, Ajit Pal Singh, Tarun Saluja, Deok Ryun Kim, Venkat Jayanth Midde, Yanghee Kim, Sridhar Vemula, Santhosh Kumar Narla, Binod Sah, Mohammad Ali

**Affiliations:** 1 Christian Medical College, Vellore, India; 2 Sanofi Pasteur, Swiftwater, Pennsylvania, United States of America; 3 Shantha Biotechnics Private Limited, Hyderabad, India; 4 International Vaccine Institute, Seoul, South Korea; 5 Johns Hopkins Bloomberg School of Public Health, Baltimore, Maryland, United States of America; Public Health England, UNITED KINGDOM

## Abstract

This open-label study assessed the safety and immunogenicity of two doses (14 days apart) of an indigenously manufactured, killed, bivalent (*Vibrio cholerae* O1 and O139), whole-cell oral cholera vaccine (SHANCHOL; Shantha Biotechnics) in healthy adults (n = 100) and children (n = 100) in a cholera endemic area (Vellore, South India) to fulfill post-licensure regulatory requirements and post-World Health Organization (WHO) prequalification commitments. Safety and reactogenicity were assessed, and seroconversion rates (i.e. proportion of participants with a ≥ 4-fold rise from baseline in serum vibriocidal antibody titers against *V*. *cholerae* O1 Inaba, O1 Ogawa and O139, respectively) were determined 14 days after each vaccine dose. No serious adverse events were reported during the study. Commonly reported solicited adverse events were headache and general ill feeling. Seroconversion rates after the first and second dose in adults were 67.7% and 55.2%, respectively, against O1 Inaba; 47.9% and 45.8% against O1 Ogawa; and 19.8% and 20.8% against O139. In children, seroconversion rates after the first and second dose were 80.2% and 68.8%, respectively, against O1 Inaba; 72.9% and 67.7% against O1 Ogawa; and 26.0% and 18.8% against O139. The geometric mean titers against O1 Inaba, O1 Ogawa, and O139 in both adults and children were significantly higher after each vaccine dose compared to baseline titers (P < 0.001; for both age groups after each dose versus baseline). The seroconversion rates for O1 Inaba, O1 Ogawa, and O139 in both age groups were similar to those in previous studies with the vaccine. In conclusion, the killed, bivalent, whole-cell oral cholera vaccine has a good safety and reactogenicity profile, and is immunogenic in healthy adults and children.

***Trial Registration*:** ClinicalTrials.gov NCT00760825; CTRI/2012/01/002354.

## Introduction

Cholera continues to be a significant public health problem in many developing countries; globally, there are an estimated 1.3–4 million cases and 21,000–143,000 associated deaths annually.[1, 2] The World Health Organization (WHO) considers immunization with cholera vaccines as a complementary cholera management strategy in the short-to-medium term in areas at risk or with endemic cholera, while other prevention and control strategies including improved sanitation and access to clean water are better developed.[1, 3]

SHANCHOL (Shantha Biotechnics Pvt. Ltd., India) is a bivalent (O1 and O139) inactivated whole-cell oral cholera vaccine (OCV) containing killed whole cells of *V*. *cholerae* O1 and *V*. *cholerae* O139. The vaccine resulted from a technology transfer of the oral, killed bivalent cholera vaccine from Vietnam to India, and was developed with the aim of producing a high quality OCV which could be WHO pre-qualified. A two-dose regimen of this vaccine was shown to be well-tolerated and immunogenic against *V*. *cholerae* O1. In a study among adults in Vietnam, the rate of vibriocidal antibody seroconversion observed was 91%. In Kolkata, India, where high background immunity exists, the observed seroconversion rates were 53% among adults and 80% among children aged ≥1 year.[4, 5] Seroconversion rates of 65% and 87% were observed in adults and children, respectively, following the first dose, and 46% and 82%, respectively, after the second dose in another study undertaken in India.[6] In addition, a large phase III cluster randomized trial conducted with 66,900 participants in India showed that two-doses of the OCV provided 65% protective efficacy against cholera for up to 5 years.[7–9]

The killed bivalent (O1 and O139) whole-cell OCV was first licensed in India in 2009 and obtained WHO prequalification in September 2011. It is also currently licensed in more than 20 countries across Asia, Africa and Latin America. Vaccine production by Shantha Biotechnics Pvt. Ltd. was fully scaled up to commercial capacity, and the present study was conducted to assess the safety and immunogenicity of the scaled-up lot of the indigenously manufactured OCV among healthy adult and children volunteers as a part of post-licensure requirements for the Indian regulatory agencies and as part of post-WHO prequalification commitments.

## Material and methods

The study was conducted at an established field site of the Department of Community Medicine, Christian Medical College (CMC), Vellore, in southern India. The trial protocol was approved by the Institutional Review Board at the Christian Medical College, Vellore, India, by the International Vaccine Institute Institutional Review Board, Seoul, Korea, and by the Indian National Regulatory Authority (DCGI) prior to the start of the trial. This study was registered with the Clinical Trials Data Bank (http://clinicaltrials.gov/), NCT Registration Identifier: 00760825, and the Clinical Trials Registry of India (CTRI/2012/01/002354).

The study conduct and selection of participants was undertaken in a similar manner as in a previous study with the vaccine.[5] In brief, healthy adults aged 18–40 years were recruited and assessed initially, and their safety data reviewed by an independent Data Safety Monitoring Board (DSMB) before proceeding with the recruitment and assessment of children aged 1–17 years. Written informed consent/assent was obtained before enrollment from adults and children aged 12–17 years, as well as written informed consent from the children’s parents/legally acceptable representative guardians. Exclusion criteria included ongoing serious chronic illness, acute illness in the preceding week, diarrhea lasting for more than 2 weeks in the previous 6 months, receipt of bacterial enteric vaccine in the last 4 weeks, or previous cholera vaccination. Pregnancy as well as abdominal pain, vomiting, loss of appetite, generalized ill-feeling or nausea during the preceding 24 hours, or diarrhea or use of anti-diarrheal or antibiotic medication during the past week were also reasons for exclusion.

### Study design

This study was an open-label, post-licensure, mono-center trial in healthy adults aged 18–40 years and children aged 1–17 years. A placebo-controlled study design was not planned due to ethical considerations as the vaccine was already licensed in India.

### Study agent and administration

The study participants received two oral doses of OCV taken 14 days apart (with a +2-day window). Each vaccine dose (1.5 ml) contained 300 ELISA Units (EU) of lipopolysaccharide (LPS) of heat-killed *V*. *cholera* O1 Inaba, classical biotype (Cairo 48); 600 EU LPS of formalin-killed *V*. *cholerae* O1 Inaba, El Tor biotype (strain Phil 6973); 300 EU LPS of heat-killed *V*. *cholerae* O1 Ogawa classical biotype (Cairo 50); 300 EU LPS of formalin killed *V*. *cholerae* O1 Ogawa classical biotype (Cairo 50); and 600 EU LPS of formalin killed *V*. *cholerae* O139 (4260B). The batch used in the study was tested and cleared by the Central Drug Laboratory at Kasauli, India, prior to use. The vaccine was supplied in single-dose vials and stored at 2–8°C until administration.

The vaccine vial was gently shaken to ensure a homogenous suspension before being drank by the recipient.

### Outcomes

Safety was assessed by monitoring solicited or unsolicited adverse events (AEs) during the study. Immunogenicity was assessed by serum vibriocidal assay, and described using seroconversion rates against O1 and O139 after the first and second vaccine dose. Additional immunogenicity analyses were performed to describe the serum vibriocidal antibody geometric mean titers (GMTs) at baseline and at day14 after each vaccine dose, as well as the geometric mean fold (GMF) rises relative to baseline.

#### Adverse event (AE) monitoring

Participants were monitored for 30 minutes after each vaccine dose by the study nurses and physicians for the occurrence of any immediate AEs. After each vaccination thereafter, the participants were followed up on an out-patient basis for three days for solicited and unsolicited AEs. A solicited AE was an adverse reaction known to occur due to the study agent and prelisted in the case report form and the diary card (DC) for the participant to record over a pre-defined period post-vaccination. An unsolicited AE was an observed AE that did not fulfill the conditions prelisted in the case report form in terms of symptom and/or onset post-vaccination. The participants or their parents were provided with a DC, with instructions on how to record solicited and unsolicited AEs, and the severity of the AEs for the next three days after each vaccination dose. The solicited events prelisted in the case report form and the DC for the study were headache, vomiting, nausea, abdominal pain/cramps, diarrhea, fever, loss of appetite, and general ill feeling. The participants attended the study center 14 days after each vaccination dose and any AEs that occurred since the previous visit were also recorded. The reported AEs were graded by the participants or their parents/guardians as mild (no interference with daily activity), moderate (some interference with daily activity) or severe (significant, prevented daily activity). Information on serious adverse events (SAEs) was collected and assessed throughout the trial, from inclusion until 14 days after the last vaccination (until Day 28). A SAE was any untoward medical occurrence that at any dose: resulted in death, was immediately life-threatening, resulted in persistent or significant disability/incapacity, required inpatient hospitalization or prolongation of existing hospitalization, a congenital anomaly/birth defect or any other medically important condition that required intervention to prevent one of the above criteria.

#### Assessment methods for determining immune responses

Serum samples were obtained from study participants prior to immunization and at the following time points: 14 days after first dose but prior to administration of the second dose and 14 days after second dose. The samples were shipped to NICED, Kolkata, maintaining appropriate cold chain, for vibriocidal antibody assessment. Vibriocidal antibody specific responses against *V*. *cholerae* O1 Inaba (strain T19479), Ogawa serotypes (strain X25049) and O139 serotypes (strain CIRS 134) were assessed using the microtiter technique as previously described.[6, 10, 11] The vibriocidal titer was defined as the reciprocal of the highest dilution that completely inhibited bacterial growth. Seroconversion was defined as a 4-fold or greater increase in the titer from baseline to post-vaccination.

### Sample size

The sample size was arbitrary agreed following discussions with DCGI and the WHO to include 200 participants, comprising 100 adults and 100 children.

### Statistical analyses

Vibriocidal antibody titers were expressed as GMTs, and GMF-rises in serum titers were also determined. Baseline versus post-immunization GMTs after each vaccine dose were compared. GMF-rises after first versus after second dose were also compared. GMT and GMF-rise were compared using paired T-test or Wilcoxon signed rank test, as appropriate, with the Shapiro-Wilk tests for normality. The seroconversion rate was defined as percentage of participants with at least a four-fold rise in serum vibriocidal antibody titers from baseline to post-vaccination. Seroconversion rates after first versus after second dose were compared. The number and percentage of participants who exhibited seroconversion after vaccination was also compared with historical immunogenicity data available for the vaccine from phase II/III studies conducted in India.[5, 6] Seroconversion rates were compared using the chi-square test with Yates correction or the Fisher’s exact test if the numbers were sparse, and the statistical significance (p-value) was derived using the exact McNemar test of marginal homogeneity. Analyses were performed in SAS 9.4 (SAS Institute, Cary, NC, USA).

## Results

### Participant flow and recruitment

**Fig 1:**
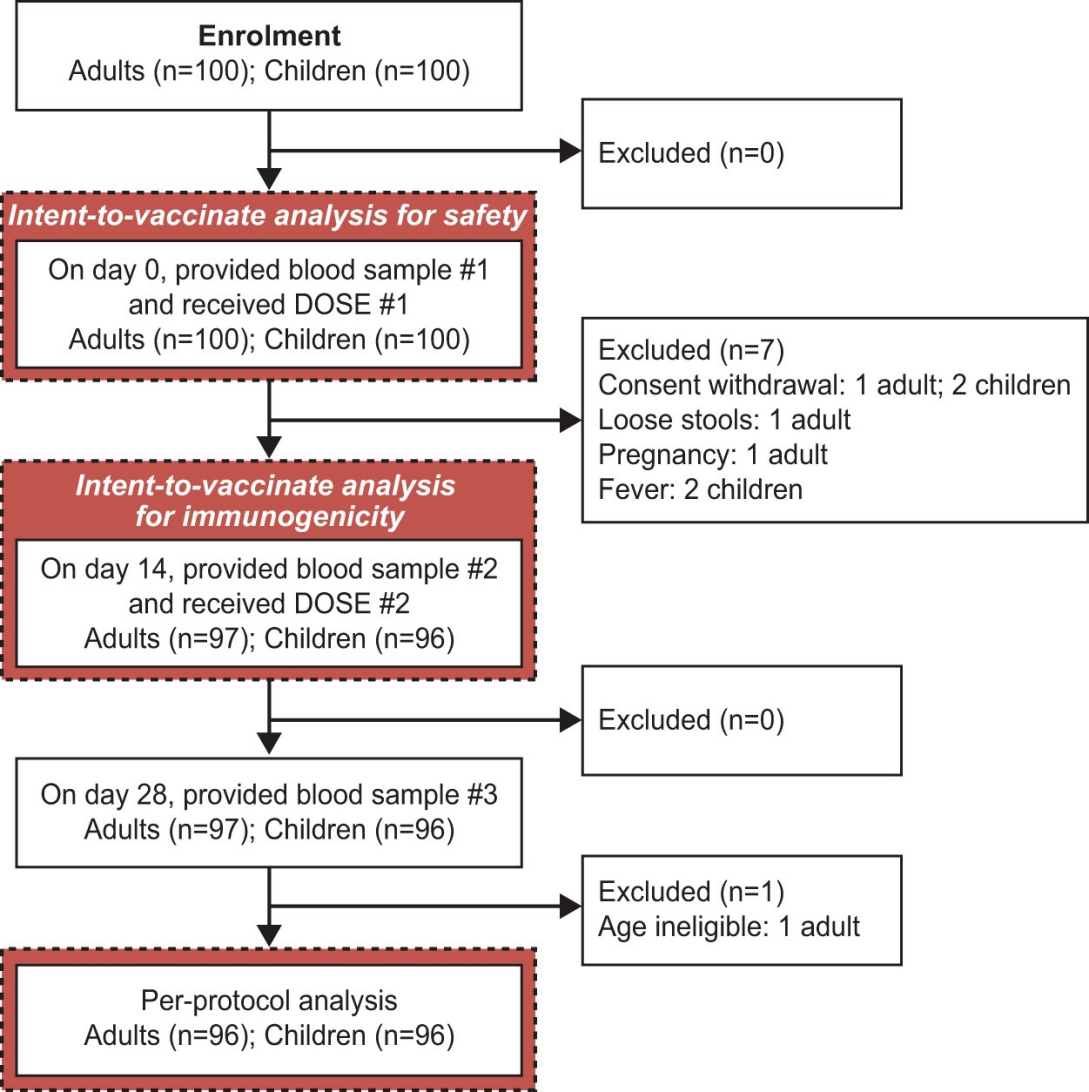
Summary of the flow of participants through the study.

Adult participants were recruited during the period March to April 2012 followed by recruitment of children from October to December 2012. The flow of adult and children participants through the study is shown in **Fig 1**.

Three adult participants discontinued the study prior to administration of the second vaccine dose. One participant withdrew consent. One participant experienced mild gastroenteritis (abdominal pain and loose stools) during the study one day prior to second dose vaccination visit (Day 13), which resolved by the next day, the time of the Day 14 visit. This participant was withdrawn from the study on the Day 14 visit, and did not receive the second vaccine dose. The third participant to discontinue was a 23 year-old female who was found to be pregnant on the day of the second study visit; she did not report missing any menstrual periods on the day of the first study visit and received the first vaccine dose. She was withdrawn from the study on the day of the second study visit when the pregnancy was identified and did not receive the second vaccine dose. She was followed-up regularly throughout her pregnancy; she delivered a baby girl weighing 3.1 kg by normal vaginal delivery at full term. Both the mother (participant) and the baby were in good health and were discharged from the primary health center on the second day.

Four children discontinued from the study prior to administration of the second vaccine dose. Consent was withdrawn for two children. One child had mild fever (38.4°C) at the second dose vaccination visit (Day 14). This child was not given the second vaccine dose at this visit and was told to come the following day after the fever had resolved but the participant did not return for vaccination. The fourth child had a mild rash and viral upper respiratory tract infection with moderate fever (38.6°C) at the time of the second dose vaccination visit (Day 14). The participant was withdrawn from the study and did not receive the second vaccine dose. The child’s symptoms resolved three days later.

### Baseline demographic data

The demographics of the participants enrolled in the study are shown in **Table 1.**

**Table 1:** Participant demographics.

Characteristics		Adults (n = 100)	Children (n = 100)
Age (years)	Mean (SD)	29.6 (4.2)	10.1 (4.4)
	Median	31.0	10.4
Gender	Male (%)	37 (37)	48 (48)
	Female (%)	63 (63)	52 (52)

### Numbers analyzed

The safety analysis was undertaken on the intention-to-vaccinate set defined as all participants enrolled who received at least one dose of study vaccine, and comprised 100 adults and 100 children. The immunogenicity analysis was performed on the per-protocol set defined as follows: enrolled, eligible participants, who received two doses of the study vaccine, were available until the last follow-up visit and provided the three blood samples as planned. In addition to the above, three adults and four children participants who discontinued the study, another adult participant was also excluded from the per-protocol analysis for immunogenicity: this participant was above the age criteria for the study but was enrolled inadvertently and completed the study. Overall 96 adults and 96 children were included in the per-protocol analysis of immunogenicity.

### Safety results

No SAEs were reported during the trial. The solicited and unsolicited AEs reported during the entire study are summarized in S1–S4 Tables.

Among the adults, 47 (47%) reported solicited AEs during the study. The most commonly reported solicited AEs were headache (28% [n = 28]) and general ill feeling (28% [n = 28]). Most of the AEs were mild to moderate in intensity. Only one adult participant reported an AE (abdominal cramps) that was of severe intensity and which resolved by the following day.

Among children, 13 (13%) reported solicited AEs during the study. The most commonly reported solicited AEs were general ill feeling (6% [n = 6]) and headache (5% [n = 5]). One child experienced severe headache and general ill feeling two days after the second dose (on Day 16) which resolved the following day.

The most commonly reported unsolicited AEs among adults were headache (9% [n = 9]), abdominal pain or cramps (7% [n = 7]) and general ill feeling (6% [n = 6]), and the most commonly reported unsolicited AEs among children were fever (6% [n = 6]), abdominal pain or cramps (4% [n = 4]) and cough (4% [n = 4]). All the unsolicited AEs reported were assessed to be unrelated or unlikely to be related to the study vaccine.

### Immune responses to *V*. *cholerae* O1 and O139

**Fig 2:**
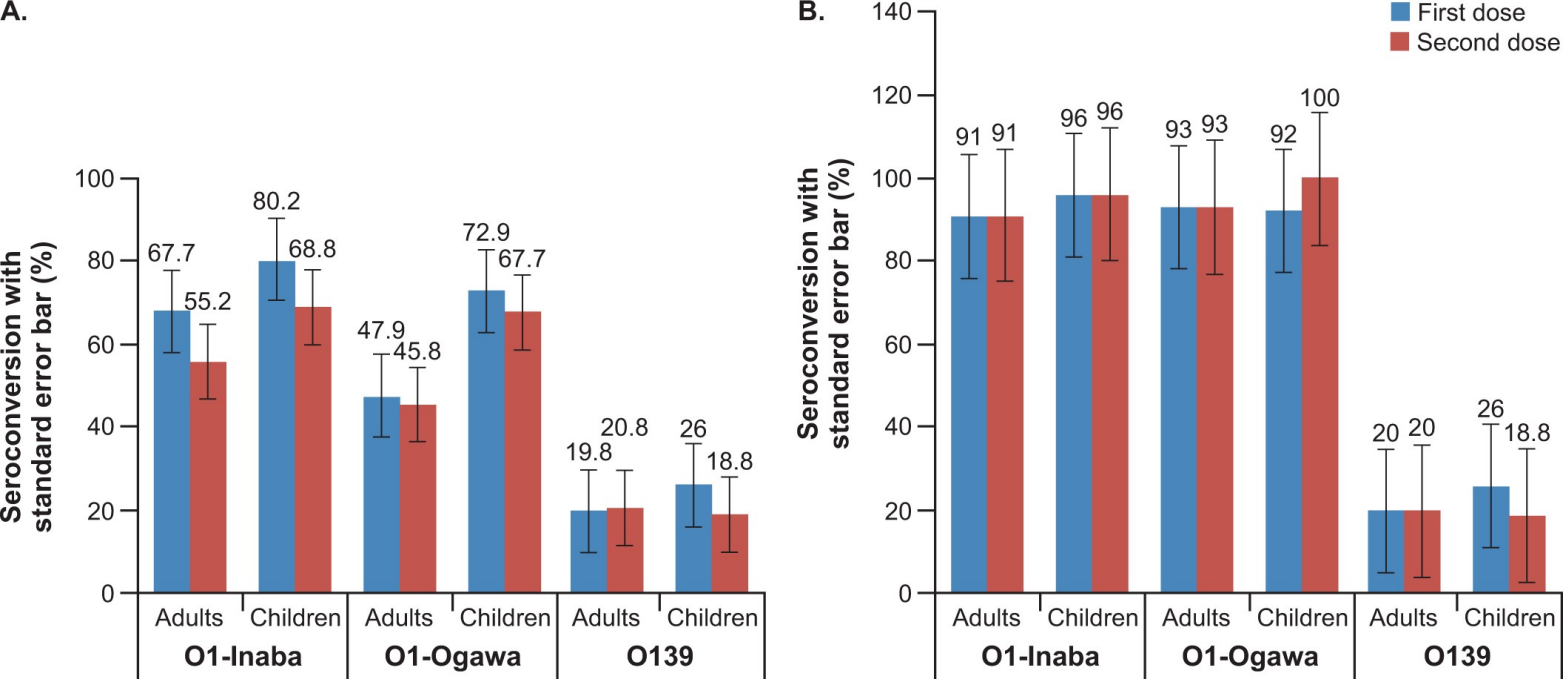
Percentage seroconversion for *V*. *cholera* O1 Inaba, *V*. *cholera* O1 Ogawa and *V*. *cholera* O139 in adults and children after one and two doses of whole-cell OCV.

The seroconversion rate after each dose of the vaccine is summarized in **Table 2 and Fig 2.**

**Table 2:** Serum vibriocidal antibody titers to *V*. *cholerae* O1 Inaba, *V*. *cholerae* O1 Ogawa and *V*. *cholerae* O139 at baseline, 14 days after the first dose and 14 days after the second dose among participants with paired blood specimens.

	*V*. *cholerae* O1 Inaba	*V*. *cholerae* O1 Ogawa	*V*. *cholerae* O139
**Adults (n = 96)**			
**No. of participants who seroconverted**^a^ **(%)**			
After dose 1	65 (67.7)	46 (47.9)	19 (19.8)
After dose 2	53 (55.2)	44 (45.8)	20 (20.8)
P-value^d^ (after dose 2 vs after dose 1)	0.004	0.625	1.000
**GMT**^b^ **(95% CI)**			
Baseline	178.3 (128.7, 247.1)	542.1 (401.6, 731.7)	5.17 (3.96, 6.74)
After dose 1	1346 (1044, 1736)	2091 (1718, 2545)	8.66 (6.39, 11.75)
After dose 2	911.7 (716.3, 1160)	1684 (1400, 2026)	7.86 (5.92, 10.44)
P-value^e^ (after dose 1 vs Baseline)	<0.001	<0.001	<0.001
P-value^e^ (after dose 2 vs Baseline)	<0.001	<0.001	<0.001
P-value^e^ (after dose 2 vs after dose 1)	<0.001	<0.001	0.060
**GMF-rise**^c^ **(95% CI)**			
After dose 1	7.55 (5.50, 10.37)	3.86 (2.87, 5.19)	1.68 (1.33, 2.11)
After dose 2	5.11 (3.86, 6.77)	3.11 (2.40, 4.03)	1.52 (1.25, 1.86)
P-value^e^ (after dose 2 vs after dose 1)	<0.001	<0.001	0.047
**Children (n = 96)**			
**No. of participants who seroconverted**^a^ **(%)**			
After dose 1	77 (80.2)	70 (72.9)	25 (26.0)
After dose 2	66 (68.8)	65 (67.7)	18 (18.8)
P-value^d^ (after dose 2 vs after dose 1)	0.007	0.227	0.065
**GMT**^b^ **(95% CI)**			
Baseline	87.87 (56.66, 136.3)	143.4 (93.83, 219.2)	3.16 (2.75, 3.62)
After dose 1	1253 (869.2, 1805)	1544 (1104, 2161)	6.38 (4.95, 8.22)
After dose 2	723.6 (529.8, 988.2)	1114 (836.7, 1484)	5.14 (4.10, 6.43)
P-value^e^ (after dose 1 vs Baseline)	<0.001	<0.001	<0.001
P-value^e^ (after dose 2 vs Baseline)	<0.001	<0.001	<0.001
P-value^e^ (after dose 2 vs after dose 1)	<0.001	<0.001	<0.001
**GMF-rise**^c^ **(95% CI)**			
After dose 1	14.25 (9.83, 20.67)	10.79 (7.56, 15.40)	2.02 (1.62, 2.52)
After dose 2	8.23 (5.86, 11.57)	7.77 (5.60, 10.77)	1.63 (1.36, 1.95)
P-value^e^ (after dose 2 vs after dose 1)	<0.001	<0.001	<0.001

^a^ Number of participants with ≥ 4 fold rise in titers of serum vibriocidal antibodies relative to baseline, 14 days after the first dose of vaccine and 14 days after the second dose of vaccine

^b^ Geometric mean reciprocal titers of serum vibriocidal antibodies at baseline, 14 days after dose 1, and 14 days after dose 2

^c^ Geometric mean fold (GMF-) rise in titers of serum vibriocidal antibodies between baseline, 14 days after dose 1, and 14 days after dose 2

^d^ The p-value was derived using exact McNemar test of marginal homogeniety.

^e^ The p-value was derived using paired T-test or Wilcoxon signed rank test, as appropriate. The Shapiro-Wilk tests for normality was done.

The baseline vibriocidal antibody titers for *V*. *cholerae* O1 Inaba, *V*. *cholerae* O1 Ogawa and *V*. *cholerae* O139 in adult participants ranged from 2.5 to 5120, 2.5 to 10240 and 2.5 to 960, respectively, and those in children participants ranged from 2.5 to 10240, 2.5 to 20480 and 2.5 to 60, respectively.

In both adult and child cohorts, the highest number of participants who seroconverted was seen for *V*. *cholerae* O1 Inaba and relatively fewer number of participants seroconverted for *V*. *cholerae* O139. However, the difference in seroconversion for O1 and O139 was not tested for statistical significance.

Seroconversion rates for *V*. *cholerae* O1 Inaba and *V*. *cholerae* O1 Ogawa were higher in children than in adults (**Fig 2**). However, the difference in seroconversion rates for adults and children was not tested for statistical significance. Of note, adults and children with baseline antibody titers ≤ 80 (an arbitrary cut-off value below which the antibody levels are considered as low [6, 10, 12]) had higher (above 90%) seroconversion rates for *V*. *cholerae* O1 Inaba and *V*. *cholerae* O1 Ogawa than those with baseline antibody titers > 80. The seroconversion rates among adults and children with baseline vibriocidal antibody titers ≤ 80 is shown in **Table 3 and Fig 2.**

**Table 3:** Proportion of adult and child participants with baseline antibody titers ≤ 80 who seroconverted between baseline and 14 days after the first dose of vaccine, and 14 days after the second dose of vaccine (among participants with paired blood specimens).

	No. of participants with baseline antibody titers ≤ 80	No. of participants with baseline antibody titers ≤ 80 who seroconverted^a^ between baseline & 14 days after dose 1 (%)	No. of participants with baseline antibody titers ≤ 80 who seroconverted^b^ between baseline & 14 days after dose 2 (%)
**Adults**			
***V*. *cholerae* O1 Inaba**	33	30 (91)	30 (91)
***V*. *cholerae* O1 Ogawa**	15	14 (93)	14 (93)
***V*. *cholerae* O139**	94	19 (20)	20 (20)
**Children**			
***V*. *cholerae* O1 Inaba**	48	46 (96)	46 (96)
***V*. *cholerae* O1 Ogawa**	36	33 (92)	36 (100)
***V*. *cholerae* O139**	96	25 (26)	18 (18.8)

^a^Number of participants with ≥ 4 fold rise in titers of serum vibriocidal antibodies between baseline and 14 days after dose 1

^b^Number of participants with ≥ 4 fold rise in titers of serum vibriocidal antibodies between baseline and 14 days after dose 2

**Fig 3:**
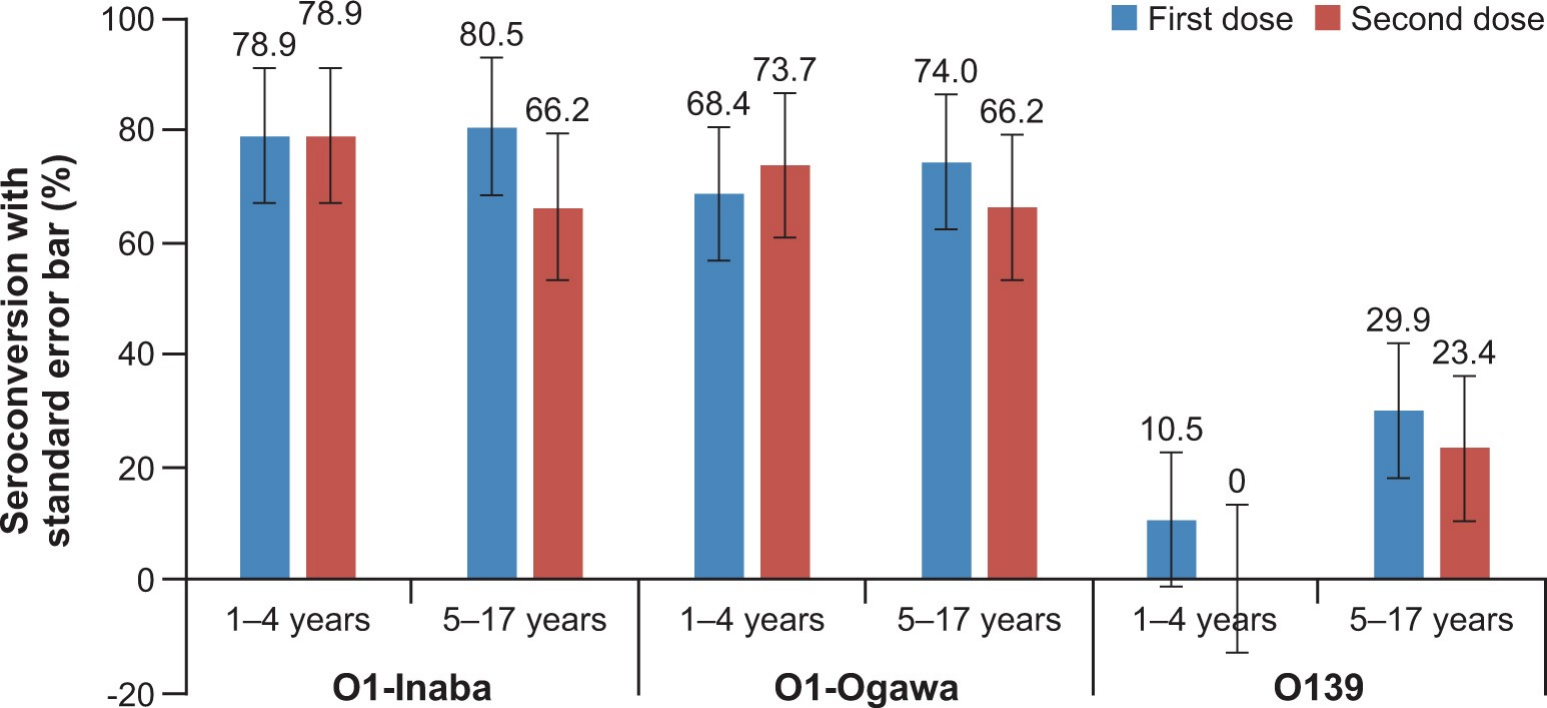
Percentage seroconversion for *V*. *cholera* O1 Inaba, *V*. *cholera* O1 Ogawa and *V*. *cholera* O139 in children aged 1–4 years and 5–17 years after receiving one and two doses of whole-cell OCV.

A higher seroconversion rate in adults and children for *V*. *cholerae* O1 Inaba, *V*. *cholerae* O1 Ogawa and *V*. *cholerae* O139 was observed after the first dose compared to that after the second vaccine dose (except for seroconversion rates in adults for *V*. *cholerae* O139) **(Fig 2A).** Similar trends were observed in children age subgroups 1–4 years and 5–17 years, except for the seroconversion rate in children aged 1–4 years for *V*. *cholerae* O1 Ogawa **(Fig 3).** However, the relatively lower seroconversion rate following the second dose compared to that after the first dose was not observed in adults and children with baseline antibody titers ≤ 80, except for the seroconversion rate in children for *V*. *cholerae* O139 **(Fig 2B).**

**Fig 4:**
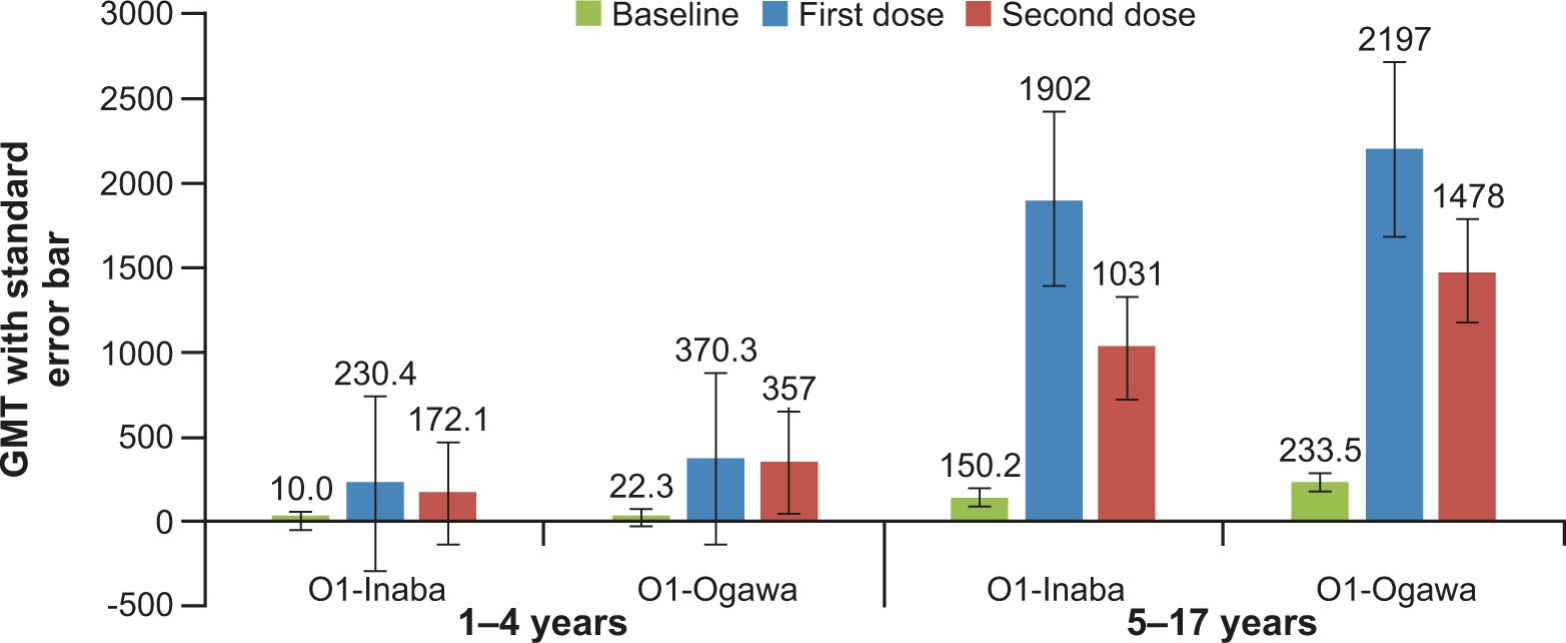
GMT for *V*. *cholera* O1 Inaba, and *V*. *cholera* O1 Ogawa in children aged 1–4 years and 5–17 years after one and two doses of whole-cell OCV.

**Fig 5:**
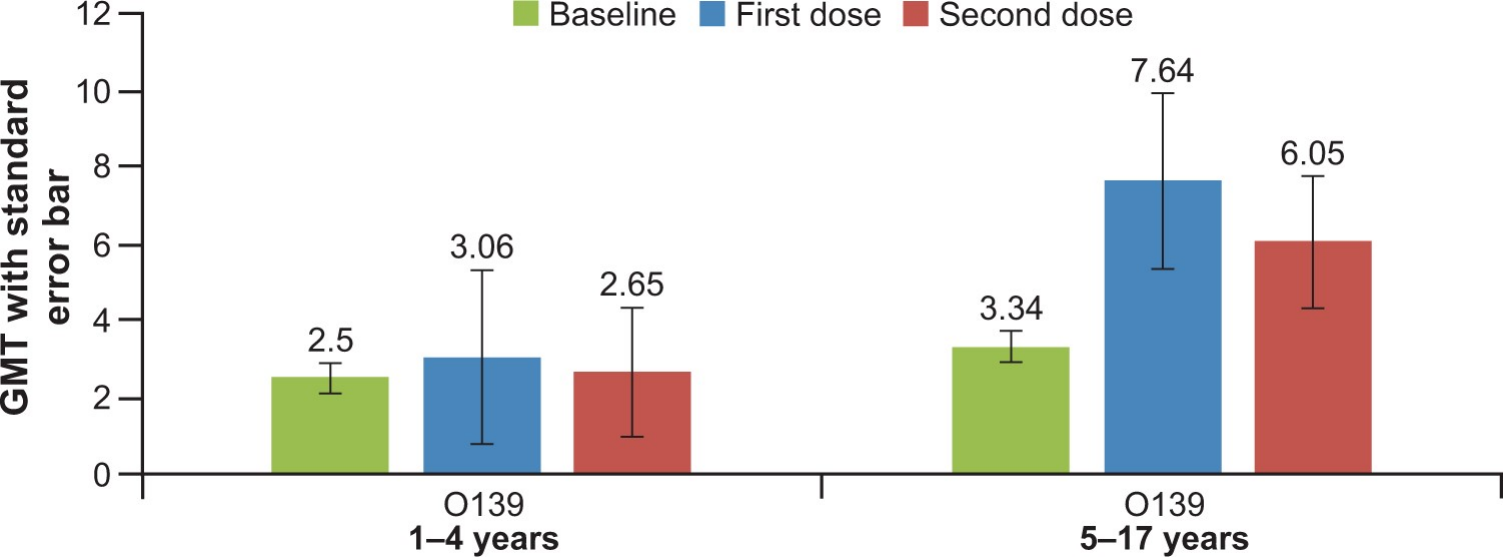
GMT for *V*. *cholera* O139 in children aged 1–4 years and 5–17 years after one and two doses of whole-cell OCV.

In both adults and children, vibriocidal GMTs for *V*. *cholerae* O1 Inaba, *V*. *cholerae* O1 Ogawa and *V*. *cholerae* O139 were significantly increased 14 days after both dose 1 and dose 2 of the vaccine, relative to baseline (P < 0.001). Again, relatively higher GMTs in adults and children for *V*. *cholerae* O1 Inaba, *V*. *cholerae* O1 Ogawa and *V*. *cholerae* O139 were observed after first dose compared to that after the second vaccine dose. The GMT results are shown in **Table 2.** A similar trend of relatively lower GMTs after the second dose compared to that after the first dose was observed in children in the age subgroups 1–4 years and 5–17 years **(Figs 4 and 5).**

**Fig 6:**
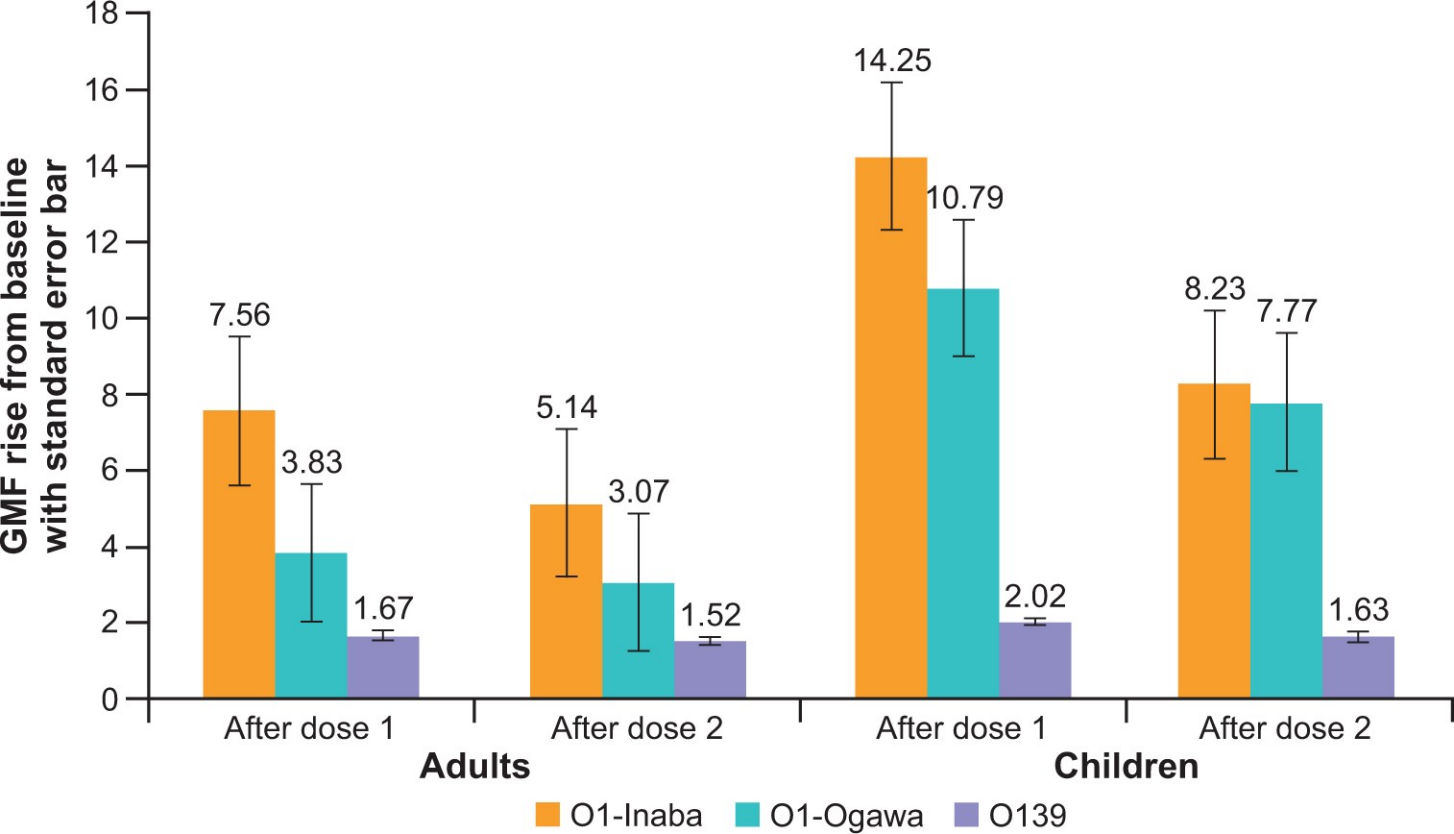
Geometric mean-fold rise in titers to *V*. *cholerae* O1 Inaba, *V*. *cholerae* O1 Ogawa and *V*. *cholerae* O139 between baseline and 14 days after the first dose of vaccine, and baseline and 14 days after the second dose of vaccine.

The GMF-rise in titres in adults and children for *V*. *cholerae* O1 Inaba, *V*. *cholerae* O1 Ogawa and *V*. *cholerae* O139 after one and two doses of the vaccine is shown in **Table 2 and Fig 6.** GMF-rise in titers for all the three serotypes were higher in children than in adults. However, the difference in GMF-rise for adults and children was not tested for statistical significance. In both adults and children, the highest GMF-rise in titers was seen with *V*. *cholerae* O1 Inaba (in adults, GMF-rise = 7.56 after first dose and 5.14 after second dose; in children, GMF-rise = 14.25 after first dose and 8.23 after second dose). The lowest GMR-rise in titers was seen with *V*. *cholerae* O139 (in adults, GMF-rise = 1.67 after first dose and 1.52 after second dose; in children GMF-rise = 2.02 after first dose and 1.63 after second dose).

## Discussion

The present study was conducted in India to evaluate the safety and immunogenicity of the fully scaled-up, indigenously manufactured, killed, bivalent (O1 and O139) whole-cell OCV in healthy adults and children residing in a cholera endemic area (Vellore in Tamilnadu, India). Similar to the previous studies conducted in Kolkata (a phase II study in 2005 and a phase III study in 2007),[5, 6] the OCV was administered as a two-dose regimen 14 days apart, and the vibriocidal antibody titers evaluated at baseline and 14 days after each dose. The indigenously manufactured OCV was found to have a good safety profile and to be immunogenic in adults and children in the present study. The commonly reported AEs were headache, abdominal pain or cramps, fever and cough. Most of the AEs were graded as mild to moderate. No SAEs were observed during the study period. The OCV safety profile in this study appears similar to that reported in other studies with both adults and children.[5, 8–10]

The present study also demonstrated that the vaccine was immunogenic in a cholera endemic population, where participants have high pre-existing vibriocidal antibody titers. Among participants with low baseline antibody titers ≤ 80, higher rates of seroconversion (over 90%) were observed for *V*. *cholerae* O1 Inaba and O1 Ogawa in both adults and children. These results show that the vibriocidal responses to OCV are inversely related to serum vibriocidal antibody titers at baseline. However, even in adults and children with baseline antibody titers ≤ 80, 9% to 7% of the participants did not achieve seroconversion. It has previously been postulated that other factors such age, nutrient deficiency or the lack of response to B cell independent carbohydrate antigens may play a role in the lower vibriocidal responses.[13, 14]

For both adults and children, the seroconversion rate was highest for *V*. *cholerae* O1 Inaba and lowest for *V*. *cholerae* O139. Even among participants with baseline antibody titers ≤ 80, the seroconversion rate was low for *V*. *cholerae* O139. The lower immune response to O139 may be related to the sensitivity of the assay used; as previously suggested, the highly diluted complement, used in the vibriocidal assay for *V*. *cholera* O1, may not be sufficient to facilitate killing of *V*. *cholerae* O139 which is capsular.[6, 15, 16]

Similar to previous studies with the OCV in cholera-endemic areas,[6, 10] higher seroconversion rates and GMTs in adults and children for *V*. *cholerae* O1 Inaba, *V*. *cholerae* O1 Ogawa and *V*. *cholerae* O139 were observed after the first dose compared to that after the second dose. The lack of increase in immune responses after the second dose has also been observed with an extended 28-day interval between the two doses in another study.[17] The higher vibriocidal response after the first dose compared to the second dose could be due to the high antigenic LPS content in the whole-cell OCV, which is about two-fold higher than that in the earlier generation oral whole-cell recombinant B subunit cholera vaccine.[4, 17, 18] It is thought that the first dose of the vaccine induces an immune response in the intestinal mucosa that blocks uptake or response to the second dose, and thus the observed lower levels after the second dose may be due to the continued waning of antibodies.[6, 17] The high baseline titers seen among the study participants suggest that they could have been previously exposed to *Vibrio cholera* infection given that the study was conducted in a cholera endemic area. It is also possible that the first dose may act like a booster in some participants given the high cholera endemicity in the region.[17] This may also explain why there was no clear trend of decreasing immune response after the second dose compared to that after the first dose in participants with low baseline titers ≤ 80, unlike those with high baseline titers.

**Fig 7:**
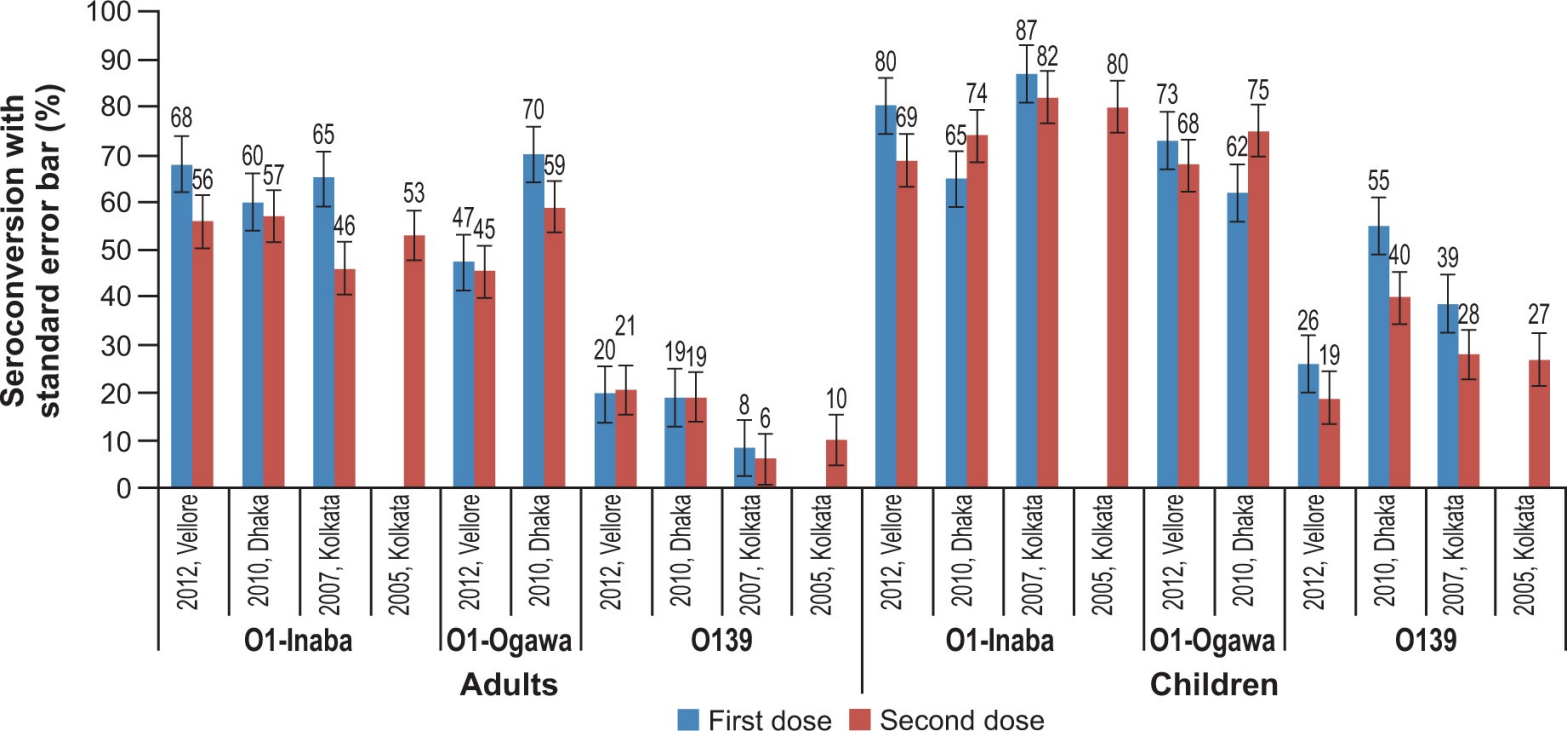
Comparison of seroconversion rates with the whole-cell OCV in studies conducted in cholera endemic areas [5, 6, 10].

The immunogenicity of OCV in this study was similar to that of other studies with the vaccine conducted in cholera endemic areas **(Fig 7)**.[5, 6] The baseline vibriocidal antibody GMTs in the present study were similar to those in other studies conducted with the vaccine in India. The baseline GMT for O1 Inaba in adults was 179 (95% CI 130 to 248) in this study compared to 251 in a previous phase II study conducted in Kolkata in 2005 [5] and 186 in a phase III study also conducted in Kolkata in 2007.[6] The differences in seroconversion rates between the present study and the previous studies in Kolkata were statistically significant only for *V*. *cholerae* O139 in adults after the second dose, with higher seroconversion rates in present study compared to that undertaken in 2007 (21% vs 6% respectively; p = 0.04). The reason for this difference may be due to optimization of the vibriocidal assay since the earlier study, and the use of lower complement concentrations.[19]

### Conclusion

As with the previous studies with whole-cell OCV, this study conducted with the fully scaled-up, indigenously manufactured vaccine demonstrated that a two-dose regimen (14 days apart) of the vaccine was well tolerated and immunogenic in adults and children aged at least 1 year.

## Supporting information

S1 Checklist TREND_Checklist_Shancol S India. (PDF)

S1 Table Solicited adverse events within 3 days following receipt of single dose or two doses. (DOCX)

S2 Table Solicited adverse events and intensity following receipt of single dose or two doses (by the number of events). (DOCX)

S3 Table Unsolicited adverse events during the entire study duration. (DOCX)

S4 Table Unsolicited adverse events during the entire study duration. (DOCX)

S1 Protocol CH-WC-02 Protocol_Version_4 0_Sep2011 (3). (PDF)
