# Supplementary material for: Safety and immunogenicity of a killed bivalent (O1 and O139) whole-cell oral cholera vaccine in adults and children in Vellore, South India
Source: PLoS One. 2019 Jun 18;14(6):e0218033. doi: 10.1371/journal.pone.0218033 (PMC6581248; doi:10.1371/journal.pone.0218033)
Supplement: S1 Table — (DOCX) [file pone.0218033.s002.docx]

**Table S1:** Solicited adverse events within 3 days following receipt of single dose or two doses

| **Solicited adverse events** | **Adult Vaccine Recipients** | | | | **Children Vaccine Recipients** | | |
| --- | --- | --- | --- | --- | --- | --- | --- |
|  | **Within 3 days after dose 1^†^**  **(n=100)** | **Within 3 days after dose 2^†^**  **(n=97)** | | **Combined AEs after dose 1 and dose 2^†^**  **(n=100)** | **Within 3 days after dose 1^†^**  **(n=100)** | **Within 3 days after dose 2^†^**  **(n=96)** | **Combined AEs after dose 1 and dose 2^†^**  **(n=100)** |
|  | **N (%)^*^** | **N (%)^*^** | | **N (%)^*^** | **N (%)^*^** | **N (%)^*^** | **N (%)^*^** |
| Diarrhea | 0 (0) | 0 | (0) | 0 (0) | 0 (0) | 0 (0) | 0 (0) |
| Abdominal pain or cramps | 5 (5) | 5 | (5.15) | 9 (9)^#^ | 2 (2.00) | 1 (1.04) | 3 (3.00) |
| Gas | 2 (2) | 0 | (0) | 2 (2) | 0 (0) | 0 (0) | 0 (0) |
| Loss of Appetite | 6 (6) | 4 | (4.12) | 9 (9)^#^ | 0 (0) | 2 (2.08) | 2 (2.00) |
| Nausea | 2 (2) | 0 | (0) | 2 (2) | 0 (0) | 0 (0) | 0 (0) |
| General ill feeling | 16 (16) | 16 | (16.49) | 28 (28)^#^ | 3 (3.00) | 5 (5.21) | 6 (6.00) |
| Fever | 1 (1) | 1 | (1.03) | 2 (2) | 1 (1.00) | 0 (0) | 1 (1.00) |
| Headache | 19 (19) | 15 | (15.46) | 28 (28)^#^ | 1 (1.00) | 4 (4.17) | 5 (5.00) |
| Vomiting | 2 (2) | 0 | (0) | 2 (2) | 0 (0) | 0 (0) | 0 (0) |
| **Total number of subjects reporting >=1 AEs** | **30** (30) | **27** | (27.84) | **47** (47)^#^ | **5** (5) | **10** (10.42) | **13** (13) ^#^ |

**^†^**Including within 30 minutes after dose

^*^Number of subjects and percentage proportion of subjects who reported adverse events

^#^ Some of the subjects reported AEs after both dose 1 and dose 2
