# Supplementary material for: Safety and immunogenicity of a killed bivalent (O1 and O139) whole-cell oral cholera vaccine in adults and children in Vellore, South India
Source: PLoS One. 2019 Jun 18;14(6):e0218033. doi: 10.1371/journal.pone.0218033 (PMC6581248; doi:10.1371/journal.pone.0218033)
Supplement: S2 Table — (DOCX) [file pone.0218033.s003.docx]

**Table S2.** Solicited adverse events and intensity following receipt of single dose or two doses (by the number of events)

|  | **Adults** | | | | | | | | | | **Children** | | | | | | | | | |
| --- | --- | --- | --- | --- | --- | --- | --- | --- | --- | --- | --- | --- | --- | --- | --- | --- | --- | --- | --- | --- |
|  | **Within 3 days after dose 1 (n=100)** | | | | | **Within 3 days after dose 2**  **(n = 97)** | | | | | **Within 3 days after dose 1 (n=100)** | | | | | **Within 3 days after dose 2**  **(n = 96)** | | | | |
|  | **Within 30 minutes of 1^st^ dose**  **(D0)** | **D1** | **D2** | **D3** | **Total** | **Within 30 minutes of 2nd dose**  **(D14)** | **D15** | **D16** | **D17** | **Total** | **Within 30 minutes of 1^st^ dose**  **(D0)** | **D1** | **D2** | **D3** | **Total** | **Within 30 minutes of 2nd dose**  **(D14)** | **D15** | **D16** | **D17** | **Total** |
| **Loose or liquid bowel movement** |  |  |  |  |  |  |  |  |  |  |  |  |  |  |  |  |  |  |  |  |
| **total** | **NA** | **0** | **0** | **0** | **0** | **NA** | **0** | **0** | **0** | **0** | **NA** | **0** | **0** | **0** | **0** | **NA** | **0** | **0** | **0** | **0** |
| **Abdominal pain or cramps** |  |  |  |  |  |  |  |  |  |  |  |  |  |  |  |  |  |  |  |  |
| mild | 0 | 1 | 1 | 1 | **3** | 0 | 3 | 1 | 2 | 6 | 0 | 1 | 0 | 0 | 1 | 0 | 0 | 0 | 0 | 0 |
| moderate | 0 | 2 | 2 | 1 | **5** | 0 | 1 | 1 | 0 | 2 | 0 | 1 | 0 | 0 | 1 | 0 | 1 | 1 | 0 | 2 |
| severe | 0 | 1 | 0 | 0 | **1** | 0 | 0 | 0 | 0 | 0 | 0 | 0 | 0 | 0 | 0 | 0 | 0 | 0 | 0 | 0 |
| **total** | **0** | **4** | **3** | **2** | **9** | **0** | **4** | **2** | **2** | **8** | **0** | **2** | **0** | **0** | **2** | **0** | **1** | **1** | **0** | **2** |
| **Gas** |  |  |  |  |  |  |  |  |  |  |  |  |  |  |  |  |  |  |  |  |
| mild | 0 | 1 | 0 | 1 | **2** | 0 | 0 | 0 | 0 | 0 | 0 | 0 | 0 | 0 | 0 | 0 | 0 | 0 | 0 | 0 |
| moderate | 0 | 0 | 0 | 0 | **0** | 0 | 0 | 0 | 0 | 0 | 0 | 0 | 0 | 0 | 0 | 0 | 0 | 0 | 0 | 0 |
| severe | 0 | 0 | 0 | 0 | **0** | 0 | 0 | 0 | 0 | 0 | 0 | 0 | 0 | 0 | 0 | 0 | 0 | 0 | 0 | 0 |
| **total** | **0** | **1** | **0** | **1** | **2** | **0** | **0** | **0** | **0** | **0** | **0** | **0** | **0** | **0** | **0** | **0** | **0** | **0** | **0** | **0** |
| **Loss of Appetite** |  |  |  |  |  |  |  |  |  |  |  |  |  |  |  |  |  |  |  |  |
| mild | 1 | 3 | 0 | 1 | **5** | 0 | 3 | 1 | 1 | 5 | 0 | 0 | 0 | 0 | 0 | 0 | 1 | 2 | 1 | 4 |
| moderate | 0 | 1 | 2 | 1 | **4** | 0 | 0 | 0 | 0 | 0 | 0 | 0 | 0 | 0 | 0 | 0 | 1 | 0 | 0 | 1 |
| severe | 0 | 0 | 0 | 0 | **0** | 0 | 0 | 0 | 0 | 0 | 0 | 0 | 0 | 0 | 0 | 0 | 0 | 0 | 0 | 0 |
| **total** | **1** | **4** | **2** | **2** | **9** | **0** | **3** | **1** | **1** | **5** | **0** | **0** | **0** | **0** | **0** | **0** | **0** | **0** | **0** | **0** |
| **Nausea** |  |  |  |  |  |  |  |  |  |  |  |  |  |  |  |  |  |  |  |  |
| mild | 1 | 2 | 0 | 0 | **3** | 0 | 0 | 0 | 0 | 0 | 0 | 0 | 0 | 0 | 0 | 0 | 0 | 0 | 0 | 0 |
| moderate | 0 | 0 | 0 | 0 | **0** | 0 | 0 | 0 | 0 | 0 | 0 | 0 | 0 | 0 | 0 | 0 | 0 | 0 | 0 | 0 |
| severe | 0 | 0 | 0 | 0 | **0** | 0 | 0 | 0 | 0 | 0 | 0 | 0 | 0 | 0 | 0 | 0 | 0 | 0 | 0 | 0 |
| **total** | **1** | **2** | **0** | **0** | **3** | **0** | **0** | **0** | **0** | **0** | **0** | **0** | **0** | **0** | **0** | **0** | **0** | **0** | **0** | **0** |
| **General ill feeling**  mild |  |  |  |  |  |  |  |  |  |  |  |  |  |  |  |  |  |  |  |  |
|  | 1 | 4 | 5 | 2 | **12** | 0 | 2 | 2 | 0 | 4 | 0 | 1 | 1 | 0 | 2 | 0 | 1 | 2 | 1 | 4 |
| moderate | 0 | 9 | 5 | 2 | **16** | 0 | 12 | 8 | 1 | 21 | 0 | 2 | 2 | 1 | 5 | 0 | 1 | 1 | 0 | 2 |
| severe | 0 | 0 | 0 | 0 | **0** | 0 | 0 | 0 | 0 | 0 | 0 | 0 | 0 | 0 | 0 | 0 | 0 | 1 | 0 | 1 |
| **total** | **1** | **13** | **10** | **4** | **28** | **0** | **14** | **10** | **1** | **25** | **0** | **3** | **3** | **1** | **7** | **0** | **2** | **4** | **1** | **7** |
| **Fever** |  |  |  |  |  |  |  |  |  |  |  |  |  |  |  |  |  |  |  |  |
| mild | 1 | 0 | 0 | 0 | **1** | 1 | 0 | 0 | 0 | 1 | 0 | 0 | 0 | 1 | 1 | 0 | 0 | 0 | 0 | 0 |
| moderate | 0 | 0 | 0 | 0 | **0** | 0 | 0 | 0 | 0 | 0 | 0 | 0 | 1 | 0 | 1 | 0 | 0 | 0 | 0 | 0 |
| severe | 0 | 0 | 0 | 0 | **0** | 0 | 0 | 0 | 0 | 0 | 0 | 0 | 0 | 0 | 0 | 0 | 0 | 0 | 0 | 0 |
| **total** | **1** | **0** | **0** | **0** | **1** | **1** | **0** | **0** | **0** | **1** | **0** | **0** | **1** | **1** | **2** | **0** | **0** | **0** | **0** | **0** |
| **Headache** |  |  |  |  |  |  |  |  |  |  |  |  |  |  |  |  |  |  |  |  |
| mild | 1 | 5 | 5 | 1 | **12** | 0 | 1 | 0 | 0 | 1 | 0 | 0 | 1 | 0 | 1 | 0 | 3 | 2 | 2 | 7 |
| moderate | 0 | 8 | 8 | 3 | **19** | 0 | 13 | 6 | 1 | 20 | 0 | 0 | 0 | 0 | 0 | 0 | 0 | 0 | 0 | 0 |
| severe | 0 | 0 | 0 | 0 | **0** | 0 | 0 | 0 | 0 | 0 | 0 | 0 | 0 | 0 | 0 | 0 | 0 | 1 | 0 | 1 |
| **total** | **1** | **13** | **13** | **4** | **31** | **0** | **14** | **6** | **1** | **21** | **0** | **0** | **1** | **0** | **1** | **0** | **3** | **3** | **2** | **8** |
| **Vomiting** |  |  |  |  |  |  |  |  |  |  |  |  |  |  |  |  |  |  |  |  |
| mild | 0 | 2 | 0 | 0 | **2** | 0 | 0 | 0 | 0 | 0 | 0 | 0 | 0 | 0 | 0 | 0 | 0 | 0 | 0 | 0 |
| moderate | 0 | 0 | 0 | 0 | **0** | 0 | 0 | 0 | 0 | 0 | 0 | 0 | 0 | 0 | 0 | 0 | 0 | 0 | 0 | 0 |
| severe | 0 | 0 | 0 | 0 | **0** | 0 | 0 | 0 | 0 | 0 | 0 | 0 | 0 | 0 | 0 | 0 | 0 | 0 | 0 | 0 |
| **total** | **0** | **2** | **0** | **0** | **2** | **0** | **0** | **0** | **0** | **0** | **0** | **0** | **0** | **0** | **0** | **0** | **0** | **0** | **0** | **0** |
