# Supplementary material for: Safety and immunogenicity of a killed bivalent (O1 and O139) whole-cell oral cholera vaccine in adults and children in Vellore, South India
Source: PLoS One. 2019 Jun 18;14(6):e0218033. doi: 10.1371/journal.pone.0218033 (PMC6581248; doi:10.1371/journal.pone.0218033)
Supplement: S4 Table — (DOCX) [file pone.0218033.s005.docx]

**Table S4.** Unsolicited adverse events during the entire study duration

| S.no | **Unsolicited Adverse Event in Adults** | **Adult Vaccine Recipients (n=100)** | S.no | **Unsolicited Adverse Event in Children** | **Children Vaccine Recipients (n=100)** |
| --- | --- | --- | --- | --- | --- |
|  |  | **N (%)^*^** |  |  | **N (%)^*^** |
| 1 | Back Pain | 2 (2) | 1 | Abdominal Pain or Cramps | 4 (4) |
| 2 | Loss Of Taste/Appetite | 3 (3) | 2 | Cold | 1 (1) |
| 3 | Abdominal Pain Or Cramps | 7 (7) | 3 | Cough | 4 (4) |
| 4 | Acid Peptic Disease | 1 (1) | 4 | Fever | 6 (6) |
| 5 | Blood In Stool | 1 (1) | 5 | General Ill Feeling | 2 (2) |
| 6 | Body Pain | 1 (1) | 6 | Head Injury | 1 (1) |
| 7 | Ear Pain | 1 (1) | 7 | Headache | 2 (2) |
| 8 | Fever | 2 (2) | 8 | Loss Of Appetite | 1 (1) |
| 9 | Gastroenteritis | 1 (1) | 9 | Rashes | 1 (1) |
| 10 | Giddiness | 2 (2) | 10 | Scabies | 1 (1) |
| 11 | General Ill Feeling | 6 (6) | 11 | Upper Respiratory Tract Infection | 1 (1) |
| 12 | Headache | 9 (9) | 12 | Viral Upper Respiratory Tract Infection | 1 (1) |
| 13 | Knee Joint Pain | 1 (1) |  |  |  |
| 14 | Knee Pain | 1 (1) |  |  |  |
| 15 | Myalgia | 3 (3) |  |  |  |
| 16 | Swelling At Neck Region | 1 (1) |  |  |  |
| 17 | Tooth Pain | 1 (1) |  |  |  |
| 18 | Upper Respiratory Tract Infect | 3 (3) |  |  |  |
| **total number of subjects reporting >=1 unsolicited AEs** | | **37** (37) |  | **total number of subjects reporting >=1 unsolicited AEs** | **16** (16) |

^*^Number of subjects and percentage proportion of subjects who reported adverse events
