# Supplementary material for: Safety and immunogenicity of a killed bivalent (O1 and O139) whole-cell oral cholera vaccine in adults and children in Vellore, South India
Source: PLoS One. 2019 Jun 18;14(6):e0218033. doi: 10.1371/journal.pone.0218033 (PMC6581248; doi:10.1371/journal.pone.0218033)
Supplement: S1 Protocol — (PDF) [file pone.0218033.s006.pdf]

AN OPEN LABEL POST LICENSURE TRIAL TO EVALUATE THE SAFETY AND IMMUNOGENICITY OF INDIGENOUSLY MANUFACTURED KILLED BIVALENT (O1 AND O139) WHOLE CELL ORAL CHOLERA VACCINE (SHANCHOL)

**COLLABORATORS:**	CHRISTIAN MEDICAL COLLEGE<br>Vellore 632 002, Tamil Nadu State India<br>Telephone 91-41-6421-4207<br>SHANTHA BIOTECHNICS PVT.LTD., Vasantha Chambers, 4th Floor, Fateh Maidan Road, Basheer Bagh, Hyderabad 500 004 India, Telephone 91-40- 66301000, Fax 91-40-23234133<br>INTERNATIONAL VACCINE INSTITUTE, SNU Research Park, San 4-8 Bongcheon 7-dong, Kwanak, Seoul, 151-818 Korea Telephone (82-2) 872-2801, Fax (82-2) 872-2803
**INVESTIGATIONAL PRODUCT:**	Shanchol [Killed Bivalent (O1 and O139) Whole Cell Oral Cholera Vaccine]
**PRINCIPAL INVESTIGATOR**:	Dr. Venkat Raghava, Christian Medical College, Vellore
**CO-INVESTIGATORS :**	Dr. Gagandeep Kang, Dr. Vinohar Balraj, Dr. Jayaprakash Muliyil, Christian Medical College, Vellore
**IVI REPRESENTATIVES:**	Dr. John D. Clemens, Mr. Rodney Carbis, Ms Sunheang Shin,<br>Dr. Thomas Wierzba
**SHANTHA REPRESENTATIVES:**	Dr. Mandeep Singh Dhingra, Dr. Tarun Saluja
**DATA MANAGEMENT:**	Dr. Mohammad Ali, Mr. Mahesh K. Puri
**STATISTICIANS:**	Ms. Deok Ryun Kim
**BIOLOGICAL LABORATORY:**	Dr. Seung Hyun Han, Dr. Stephen Attridge
**DATA SAFETY MONITORING BOARD (DSMB):**	Dr. Bernard Ivanoff, Dr. Ira Longini, Dr. Taesun Park
**NCT REGISTRATION:**	00760825
**PROTOCOL NUMBER:**	CH-WC-02
**VERSION NUMBER//DATE:**	Version 4.0, September 2011

TABLE OF CONTENTS

1 INTRODUCTION 5
1.1 Background 5
1.2 Study Rationale 7
2 STUDY OBJECTIVES 7
2.1 Primary objectives: 7
3 STUDY DESIGN 7
3.1 Endpoints 7
3.1.1 Primary Endpoints 7
3.1.2 Secondary Endpoints 8
3.2 Type of study 8
4 STUDY SUBJECTS 8
4.1 Recruitment 8
4.2 Inclusion Criteria 9
4.3 Exclusion Criteria 9
4.4 Subject withdrawal during the study 9
4.5 Identification of subjects 10
4.6 Duration of the study period for one subject 10
5 STUDY AGENTS 10
5.1 Vaccine 10
5.2 Packaging and coding 11
5.3 Investigational product accountability 11
5.4 Storage conditions 11
5.5 Administration 11
6 6. STUDY PROCEDURES 12
6.1 Schedule and description of observations and visits 12
6.2 Assessment after vaccination 12
6.2.1 Follow-up for Adverse Events 12
6.2.2 Follow-up for Serious Adverse Events 13
6.2.3 Blood Draws 13
6.2.4 Laboratory procedures 14
7 ADVERSE EVENTS 14
7.1 Definitions 14
7.2 Assessment of Causality 15
7.3 Assessment of Severity 16
7.4 Reporting of Serious Adverse Events 17
8 DATA COLLECTION AND MANAGEMENT PROCEDURES 17
8.1 Case Report Forms (CRF) 17
8.2 Source Documentation 17
8.3 Data Management 18
9 STATISTICAL CONSIDERATIONS 18
9.1 Sample Size Calculation 18
9.2 Analysis Plans 19
10 MONITORING, AUDITING, INSPECTION 20
10.1 Responsibilities of the investigator(s) 20
10.2 Responsibilities of the IVI coordinators and Shantha Monitors 20
10.3 Responsibilities of the DSMB 20
11 ETHICAL AND REGULATORY STANDARDS 21
11.1 Ethical principles/ Laws and regulations 21
11.2 Potential risks and risk minimization 21
11.3 Informed consent and Assent 21
11.4 Institutional Review Committee (IRB) / Ethics Committee (EC) 22
12 ADMINISTRATIVE ASPECTS 22
12.1 Record retention 22
12.2 Publications 23
12.3 Study Funding 23

List of Abbreviations

AE	Adverse event
CI	Confidence Interval
CMC	Christian Medical College
CRF	Case Report Form
DSMB	Data Safety and Monitoring Board
EC	Ethics Committee
EU	Enzyme Linked Immunosorbent Assay (ELISA) Units
ICH – GCP	International Conference on Harmonization- Good Clinical Practices
GMP	Good Manufacturing Practices
GMT	Geometric Mean Titers
IRB	Institutional Review Board
IVI	International Vaccine Institute
LPS	Lipopolysaccharide
NIHE	National Institute of Hygiene and Epidemiology
NICED	National Institute of Cholera and Enteric Diseases
OCV	Oral Cholera Vaccine
SAE	Serious Adverse Event
VABIOTECH	Company for Vaccine and Biological Production No. 1
WHO	World Health Organization

STUDY SUMMARY

**TITLE**	An Open label post licensure trial to evaluate the safety and immunogenicity of indigenously manufactured Killed Bivalent (O1 and O139) Whole Cell Oral Cholera Vaccine (Shanchol)
**STUDY OBJECTIVES**	**Primary objectives:**<br>1. To confirm the safety of the killed bivalent oral cholera vaccine (Shanchol) produced by Shantha Biotechnics in healthy adult and children volunteers.<br>2. To determine the immune responses to the killed bivalent oral cholera vaccine (Shanchol) produced by Shantha Biotechnics among healthy adult and children volunteers.
**STUDY DESIGN**	Open label trial in healthy adults and children allocated to receive two administrations of the vaccine.
**STUDY SUBJECTS**	Healthy, non pregnant adults aged 18 – 40 years and<br>Healthy children aged 1 – 17 years.
**SAMPLE SIZE**	200 subjects, 100 adults and 100 children
**STUDY AGENT**	• Killed bivalent (O1 and O139) whole cell oral cholera vaccine (Shanchol)
**STUDY END-POINTS**	**Primary:**<br>1. Safety: Proportion of subjects with diarrhea<br>2. Immunogenicity: Proportion of subjects exhibiting 4-fold or greater rises in titers of serum vibriocidal antibodies, relative to baseline, 14 days after the first dose and 14 days after the second dose of vaccine.<br>**Secondary:**<br>1. Geometric mean serum vibriocidal titers at baseline, 14 days after dose 1, and 14 days after dose 2 of killed oral cholera vaccine.<br>2. Proportion of subjects given killed oral cholera vaccine with any of the following adverse events:<br>a. Immediate reactions within 30 minutes after each dose<br>b. Serious Adverse Events occurring throughout the trial<br>c. Reactogenicity: Headache, vomiting, nausea, abdominal pain/cramps, gas, diarrhea, fever, loss of appetite, general ill feeling<br>i. Diarrhea is defined as having 3 or more loose/watery stools within a 24 hour period.<br>ii. Fever is defined as having an oral temperature of $\geq 38^{o}$ C or axillary temperature of $\geq 37.5^{o}$ C
**STUDY PERIOD**	6 months

FLOWCHART (Schedule of Visits)

	Visit Day								
**Screening and Clinical Exam**	0	1	2	3	14	15	16	17	28
Informed Consent	X								
History and Physical Exam	X								
Screening	X								
Randomization	X								
Clinical Evaluation					X				
**Vibriocidal Assay**									
Blood Draw- Vibriocidal Assay	X				X				X
**Administration of Study Agent**									
Administration of Study Agent- Killed Oral Cholera Vaccine	X				X				
Solicited Symptoms	X	X	X	X	X	X	X	X	X

1 INTRODUCTION

1.1 Background

Cholera re-emerged as a global threat to public health in 2006 with a 79% increase in cases compared to the previous year. With increasing numbers of displaced populations living in unsanitary conditions and improvements to sanitation and hygiene unrealistic in the near future, the problem is likely to continue escalating. Approximately 99% of the reported cholera cases and most of the cholera deaths were from Africa. The case fatality rate (CFR) in Africa was as high as 30% in some high risk areas, and the worldwide CFR increased to 2.7% from 1.7% in 2005.[1] These figures are believed to be underestimates, with as many as 1 million cases and 100 000 to 130 000 deaths believed to occur each year.[2] Underreporting is assumed to exist because of inconsistencies in the definition of cholera, limitations of surveillance, and fear of international travel and trade sanctions. The revised International Health Regulations have been in effect since June 2007 to ensure that trade and economic sanctions will no longer be imposed on countries with cholera. Instead, open reporting of cholera outbreaks is encouraged so that they can be contained in a timely manner.[3]

In 2006, India reported 1939 cases of cholera to the WHO [1] with states such as West Bengal, Maharashtra, Andhra Pradesh, Tamil Nadu, Karnataka and Delhi long been reporting outbreaks.[4-7] Since the dramatic appearance of the new serotype O139 Bengal in 1992 in Madras[8], both *V cholerae* O1 and O139 are present in India.

Provision of safe water and food, establishment of adequate sanitation, and implementation of personal and community hygiene constitute the main public health interventions against cholera. These measures cannot be implemented fully in the near future in most cholera-endemic areas. Improvements to water and sanitation require substantial long-term investments, commitment from the local government, and often take years to implement. In the meantime, a safe, effective and affordable vaccine would be a useful tool for cholera prevention and control.[9]

Considerable progress has been made during the last decade in the development of new generation oral vaccines against cholera. These have already been licensed in some countries and are now being considered for wider public health application.[9] Cholera immunization is now recommended for travelers to high risk areas, refugee camps and for outbreak response. Furthermore, expanded use of cholera vaccines may be recommended for endemic areas, where there is increasing demand from both low- and middle-income populations.

A monovalent (anti-O1) oral killed cholera vaccine was developed by Prof Jan Holmgren in Sweden and is now licensed to a pharmaceutical company in the United Kingdom. The vaccine consists of inactivated whole cells of *V. cholerae* supplemented with a purified recombinant–DNA derived B-subunit of the cholera toxin. Large scale field trials of the vaccine in Bangladesh and Peru showed that both the killed whole cell vaccine containing the B-subunit, as well as the killed whole cell preparation alone, conferred significant protection for recipients for up to 3-5 years depending on age. An initial protection of 85-90% was obtained with the killed vaccine containing the B-subunit but this level of protection declined to about 50% after 6 months. The oral vaccine

lacking the B-subunit gave a somewhat lower initial level of protection but after 6 months the protection afforded by the two vaccines was similar.[10-11] The vaccine is licensed in several industrialized countries and is used mainly by travelers. Unfortunately, the vaccine is prohibitively expensive for public health use in developing countries.

Starting in the mid-1980s, following technology transfer from Prof Jan Holmgren, Vietnamese scientists at the National Institute of Hygiene and Epidemiology (NIHE) in Hanoi developed and produced an oral, killed cholera vaccine for the country's public health programs. A two-dose regimen of a first generation monovalent (anti-O1) cholera vaccine produced at US$ 0.10 per dose underwent a field trial in Hue, Vietnam.[12] The study was not formally randomized: the vaccine was assigned on the basis of a systematic allocation scheme and the control group did not receive a placebo. The calculated efficacy against El Tor cholera was 66% in fully immunized adults and children. Protection against non-cholera was assessed and none was found suggesting a non-biased study design. Subsequently, killed 0139 whole cells were added to the Vietnamese vaccine due to the emergence of the new form of epidemic cholera caused by this serogroup. A study found the bivalent vaccine to be safe and immunogenic in adults and children one year and older.[13]

The Vietnamese vaccine has several distinct advantages over the Swedish vaccine. The Vietnamese vaccine confers protection against the El Tor biotype in younger children. And the price of US $0.10 per dose is feasible for public health programs in developing countries, while the Swedish vaccine is prohibitively expensive. Finally, it can be administered without a buffer, while the Swedish vaccine requires a buffer and stricter cold chain requirements.

Since licensure of the oral cholera vaccine in Vietnam, more than 9 million doses have been administered without any report of serious adverse events. The vaccine is produced according to recommended guidelines at the Company for Vaccine and Biological Production No. 1 (VABIOTECH) in Hanoi. VABIOTECH is working towards WHO Good Manufacturing Practices (GMP) certification, which they hope to receive in the next few years. At the same time, the IVI and VABIOTECH have been working to internationalize the Vietnamese vaccine for global use. In order to comply with WHO requirements, the vaccine was reformulated.

Phase II trials of this reformulated killed oral cholera vaccine were performed in SonLa, Vietnam and Kolkata, India where the vaccine was found to be safe and no serious adverse reaction was associated with the vaccine. The vaccine elicited significant vibriocidal antibody responses among vaccinees. In SonLa, 90% of adult recipients seroconverted to *V. cholerae* O1 following receipt of two doses of the vaccine. In Kolkata, 53% of adults and 80% of children aged 1-17 years developed 4-fold and greater rises in vibriocidal antibodies to *V. cholerae* O1. Data from Vietnam and India suggest that greater magnitudes in the vibriocidal responses following 2 doses of the vaccine are elicited compared to previous formulations.[14-15] It has been suggested that this response may correlate with the higher lipopolysaccharide content of the vaccine, a result of changes in its standardization.[13]

A phase III trial of this vaccine was carried out in Wards 29, 30, and 33 of Kolkata, India. The vaccine demonstrated a 67% efficacy against choler caused by V. cholerae O1 even after two years of vaccination. There was no statistically significant difference between the adverse event profiles

of the vaccine and the placebo groups. The follow up of this trial is ongoing to evaluate the efficacy after three years of vaccination.[16]

Through an agreement negotiated by the IVI, VABIOTECH produced the bulk reformulated bivalent vaccine under quality conditions supervised by the IVI. Shantha Biotechnics of India filled and finished the bulk, and obtained regulatory clearance for use of the vaccine in Phase II and III trials in India. In return, the technology for future production of the oral killed bivalent cholera vaccine was transferred to Shantha Biotechnics.

Vaccine production by Shantha Biotechnics is fully scaled up, and the vaccine is available for testing. Data regarding the safety and immunogenicity of this Indian produced vaccine is necessary to proceed with an application for licensure in India. With licensure from the national regulatory authority which has been pre-qualified by WHO, the vaccine would have the potential to reach cholera-endemic areas worldwide.

1.2 Study Rationale

A study is necessary in order to assess the safety and immunogenicity of the indigenously manufactured bivalent killed whole cell oral cholera vaccine produced in India by Shantha Biotechnics among healthy adult and children volunteers. Along with the results of the phase III trial, the results of this study will help pave the way for introduction of the vaccine in India as well as other countries where cholera continues to be a major threat to public health.

2 STUDY OBJECTIVES

2.1 Primary objectives:

1. To confirm the safety of the oral killed bivalent cholera vaccine produced by Shantha Biotechnics in healthy adult and children volunteers.

2. To determine the immune responses to the oral killed bivalent cholera vaccine produced by Shantha Biotechnics among healthy adult and children volunteers.

3 STUDY DESIGN

3.1 Endpoints

3.1.1 Primary Endpoints

The primary endpoints of the study are as follows:

1. Safety:

    Proportion of subjects with diarrhea

2. Immunogenicity:

    Proportion of subjects exhibiting 4-fold or greater rises in titers of serum vibriocidal antibodies, relative to baseline, 14 days after the first dose of vaccine and 14 days after the second dose of vaccine.

3.1.2 Secondary Endpoints

1. Geometric mean serum vibriocidal titers at baseline, 14 days after dose 1, and 14 days after dose 2 of killed oral cholera vaccine (Shanchol).

2. Proportion of subjects given killed oral cholera vaccine with any of the following adverse events:
    a. Immediate reactions within 30 minutes after each dose
    b. Serious Adverse Events occurring throughout the trial
    c. Reactogenicity: Headache, vomiting, nausea, abdominal pain/cramps, gas, diarrhea, fever, loss of appetite, general ill feeling

        i. Diarrhea is defined as having 3 or more loose/watery stools within a 24 hour period.
        ii. Fever is defined as having an oral temperature of $\geq 38^{o}$ C or axillary temperature of $\geq 37.5^{o}$ C

3.2 Type of study

This is an open label trial among healthy adults (aged 18 – 40 years) and children (aged 1 – 17 years) who would receive 2 doses of Shanchol, the killed bivalent whole cell oral cholera vaccine

4 STUDY SUBJECTS

The target enrolment is 200 subjects, 100 adults and 100 children.

4.1 Recruitment

The study will be conducted in an established CMC field site in Vellore. Field workers in the site visit community members regularly. During these home visits, the field workers will identify individuals who may be interested in participating and invite them to visit the field clinic.

4.2 Inclusion Criteria

Healthy adults aged from 18 – 40 years and healthy children aged 1 - 17 will be recruited in Vellore.

All subjects must satisfy the following criteria at study entry:

1. Male or female adults aged 18-40 years and children aged 1 -17 years who the investigator believes will comply with the requirements of the protocol (i.e. available for follow-up visits and specimen collection).
2. For females of reproductive age, they must not be pregnant (as determined by verbal screening).
3. Written informed consent obtained from the subjects or their parents/guardians, and written assent for children aged 12 – 17 years.
4. Healthy subjects as determined by:
   - Medical history
   - Physical examination
   - Clinical judgment of the investigator

4.3 Exclusion Criteria

The following criteria should be checked at the time of study entry, if any of the following is present then the subject will be excluded from the study:

1. Ongoing serious chronic disease
2. Immunocompromising condition or therapy
3. Diarrhea (3 or more loose/more watery stools within a 24-hour period) 6 weeks prior to enrollment
4. One or two episodes of diarrhea lasting for more than 2 weeks in the past 6 months
5. One or two episodes of abdominal pain lasting for more than 2 weeks in the past 6 months
6. Intake of any anti-diarrhea medicine in the past week
7. Abdominal pain or cramps, loss of appetite, nausea, general ill-feeling or vomiting in the past 24 hours
8. Acute disease one week prior to enrollment, with or without fever. Temperature ≥38ºC (oral) or axillary temperature ≥ 37.5ºC warrants deferral of the vaccination pending recovery of the subject
9. Receipt of antibiotics in past 14 days
10. Receipt of live or killed enteric vaccine in past 4 weeks
11. Receipt of killed oral cholera vaccine

4.4 Subject withdrawal during the study

Each subject is free to accept or reject the proposal to enroll in this study. Even after enrollment the subject will be able to withdraw from the study at any time.

The following criteria should be checked at each visit subsequent to the intake of vaccine:

1. Use of any immunosuppressive or immune-modifying drugs during the study period (for corticosteroids this would mean ≥0.5 mg/kg/day)

2. Administration of immunoglobulins or any blood product during the study period

If any of the above criteria is applicable, then it may affect the subject's evaluation in the per-protocol analysis, however the individual will not be withdrawn from the study.

4.5 Identification of subjects

An identification card will be provided to all study participants. Participants will be asked to bring the card whenever they return to the study center. Follow-up visit dates will be written in the identification cards.

4.6 Duration of the study period for one subject

Each study subjects' participation will last for 28 days (up to + 32 days).

5 STUDY AGENTS

5.1 Vaccine

The Killed Bivalent (O1 and O139) Whole Cell Oral Cholera Vaccine (Shanchol) will be provided by Shantha Biotechnics. Each vaccine dose (1.5 ml) contains the following:

Vaccine strain	***Concentration***
V. cholerae O1 Inaba El Tor strain Phil 6973 formalin killed	600 Elisa units (EU) of lipopolysaccharide (LPS)
V. cholerae O1 Ogawa classical strain Cairo 50 heat killed	300 EU LPS
V. cholerae O1 Ogawa classical strain Cairo 50 formalin killed	300 EU LPS
V. cholerae O1 Inaba classical strain Cairo 48 heat killed	300 EU LPS
V. cholerae O139 strain 4260B formalin killed	600 EU LPS
Thiomersal	Not more than 0.02% (w/v)
Buffer	q.s. to 1.5 mL

Previously, aliquots from the individual lots of cholera vaccine have undergone extensive quality control testing at the University of Gothenburg, and Shantha Biotechnics for sterility, detoxifying agent, thiomersal assay, immunogenicity in animals, functional residual cholera toxin activity (rabbit skin test) and LPS content by an ELISA method using polyclonal antibody.

5.2 Packaging and coding

The vaccine comes in single-dose vials containing 1.5 ml of study agent. All individual vials will be labeled to reflect the study title and the study registration number along with batch number and expiry dates.

5.3 Investigational product accountability

Complete and accurate written records of receipt and storage and utilization of the vaccine including: date received, lot number, quantity received and doses administered (with the identification of the subject) must be maintained by the site study staff. Any known discrepancies in the accountability of the vaccine must be adequately documented. At the end of the trial, the unused vaccine will be returned to Shantha Biotechnics by the investigator. The investigator will not use the vaccine in any other manner than that provided for in the protocol.

The principal investigator will obtain the necessary clearances from the appropriate local agencies for the use in clinical trial of the study agent (cholera vaccine). Shantha Biotechnics will obtain the necessary clearances from the Drug Controller General of India (DCGI) and the Central Research Institute, Kasauli. The study agents will be kept in a secure place.

5.4 Storage conditions

The study agents will be stored in a secure place between +2º to +8º C before administration.

5.5 Administration

After acquisition of informed consent and ascertainment of eligibility, consenting, eligible subjects will be entered into the trial.

At the time of the first dose, information about vaccine administration will be entered into CRF Day 0. This information will note the success of administration as well as certain additional information.

Cups or syringes used for vaccination will be disposed of after each dose.

Fourteen days (and up to 16 days) after the first dose, a second dose of the same code will be administered according to the same procedures. The only contraindications to the second dose will be:

1. The occurrence, after the first dose, of a severe allergic reaction (generalized urticaria, wheezing, anaphylaxis)
2. The development of an inter-current illness after the first dose, judged by the Principal Investigator to be too severe to continue participation

6 6. STUDY PROCEDURES

6.1 Schedule and description of observations and visits

See flowchart on page iv.

a. **<u>Day 0</u>**: Informed consent will be obtained from the subject or parent/guardian. If the subject is 12-17 years old, then assent is also obtained after the informed consent is signed (or marked with thumbprint) by the parent/guardian. Next, screening for inclusion and exclusion criteria, history and physical examination will be completed by the study physicians. Then blood will be obtained for baseline (pre-immunization) immunologic tests. The study agent (vaccine) will be given subsequently. The subjects will be asked to wait in the clinic for 30 minutes for adverse event monitoring by the study physicians. CRF Day 0 will be completed.

b. **<u>Day 1-3:</u>** Subjects are followed-up for interval solicited adverse event monitoring (either they return to the center or are visited at home). CRF Day 1-3 will be completed.

c. **<u>Day 14 (*up to 16 days after administration of dose 1*)</u>**: Subjects return to the study center for screening, interval clinical evaluation and adverse event monitoring. Blood will be obtained for testing of post-first dose immunologic response. The second dose of study agent (vaccine) is given. The subject will be asked to wait in the clinic for 30 minutes for adverse event monitoring.

d. **<u>Day 15-17</u>**: Subjects are followed-up for interval solicited adverse event monitoring (either they return to the center or are visited at home). CRF Day 15 - 17 will be completed.

e. **<u>Days 28 (*up to 16 days after administration of dose 2*)</u>**: All subjects return to the study center for interval clinical evaluation including adverse event monitoring. Blood is obtained for testing of post-second dose immunologic response. CRF Day 28 and the Study Summary are completed.

6.2 Assessment after vaccination

6.2.1 Follow-up for Adverse Events

Following each dose, subjects will be observed in the clinic (vaccinating area) for 30 minutes to assess for any immediate reactions.

After each dose of study agent (vaccine) the subjects will be followed up on an out-patient basis (either asked to return to the center or followed up at home) for three days. The subject will provide a 24-hour recall history of symptoms and the axillary body temperature will be taken. Study staff will complete the appropriate CRF pages. Any unsolicited adverse events will be documented on CRF Appendix for Adverse Events.

In addition to the three days of follow up following each dose, interval clinical evaluation will be performed on day 14 and 28 to assess for any adverse events that may have occurred. Any adverse events noted will be recorded on CRF Appendix for Adverse Events.

Medications taken, non- solicited Adverse Events and Serious Adverse Events reported during the study period will be recorded on the appropriate Appendix pages of the CRF.

6.2.2 Follow-up for Serious Adverse Events

Any serious adverse event which occurs during subject's participation in the study will be reported using the Serious Adverse Events Form.

A serious adverse event (experience) is any untoward medical occurrence that at any dose:

- Results in death,
- Is life-threatening. The term "life-threatening" in the definition of "serious" refers to an event in which the subject was at risk of death at the time of the event; it does not refer to an event, which hypothetically might have caused death, if it were more severe.
- Requires in subject hospitalization or prolongation of existing hospitalization,
- Results in persistent or significant disability/incapacity,
- Results in a congenital anomaly/birth defect, or
- Any other important medical event that may not be immediately life-threatening or result in death or hospitalization but may jeopardize the subject or may require medical or surgical intervention to prevent one of the other outcomes listed in the definition above. Prudent medical judgment must be exercised to decide whether reporting is appropriate. An example includes treatment for allergic bronchospasm that does not result in hospitalization but required intensive medical intervention in the emergency room.

Medical and scientific judgment should be exercised in deciding whether expedited reporting is appropriate in other situations, such as important medical events that may not be immediately life-threatening or result in death or hospitalization but may jeopardize the subject or may require intervention to prevent one of the other outcomes listed in the definition above. These should also usually be considered serious.

6.2.3 Blood Draws

Venepuncture will be performed prior to dose 1, 14 days after dose 1, and 14 days after dose 2 for the 200 subjects enrolled in the study. Each specimen will be labeled with the following information: date of blood draw, initials, and study ID number.

After the first bleed, the CRF Day 0 will be completed to indicate the success of the blood collection. CRF Day 14 and CRF Day 28 will be completed after the second and third bleeds, respectively. For each blood draw, numbered laboratory stickers will be affixed to the corresponding CRF and specimen.

6.2.4 Laboratory procedures

Serum vibriocidal antibody assay

The vibriocidal antibody assay is a bactericidal assay requiring the presence of complement-fixing antibody bound specifically to vibrios; this serum antibody response increases after clinical cholera or after vaccination. The serum samples from the volunteer prior to immunization and 14 days after each dose will be tested using vibriocidal antibody assay. An increase of titer by 4-fold or greater between baseline and post-immunization sera will be considered a significant antibody response.

The vibriocidal assay using the microtiter technique will be performed at National Institute of Cholera and Enteric Diseases (NICED), Kolkata. Samples from CMC, Vellore will be shipped to NICED, Kolkata in appropriate cold chain.

7 ADVERSE EVENTS

7.1 Definitions

ADVERSE EVENTS:

An adverse event is defined as any noxious, pathologic, or unintended change in anatomic, physiologic, or metabolic functions, as indicated by physical signs, symptoms, and/or laboratory changes occurring in any phase of the clinical trial, regardless of their relationship to study medication. Adverse events include:

- an exacerbation of a pre-existing condition
- an intercurrent illness
- any drug interaction
- any event related to a concomitant medication
- pregnancy

A treatment-emergent event is defined as any event not present prior to exposure to study medication or any event already present that worsens in either intensity or frequency following exposure to study medication.

Possible adverse events would include abdominal pain, loss of appetite, nausea, general ill feeling, fever and vomiting. Follow-up for adverse events following immunization will be conducted and recorded as described in section 6.2.

SERIOUS ADVERSE EVENTS:

A Serious Adverse Event means any event that results in:

- death
- is immediately life-threatening
- results in persistent or significant disability/incapacity
- requires inpatient hospitalization or prolongation of existing hospitalization

- is a congenital anomaly/birth defect
- Any other medically important condition that required intervention to prevent one of the above criteria.

Important medical events that may not result in death, be life-threatening, or require hospitalization may be considered a serious adverse event when, based upon appropriate medical judgment, they may jeopardize the subject and may require medical or surgical intervention to prevent one of the outcomes listed in this definition. Examples of such medical events include allergic bronchospasm requiring intensive treatment in an emergency room or at home, blood dyscrasias or convulsions that do not result in in-subject hospitalization, or the development of drug dependency or drug abuse.

7.2 Assessment of Causality

The Investigator's assessment of an adverse event's relationship to study drug is part of the documentation process, but it is not a factor in determining what is or is not reported in the study. If there is any doubt as to whether a clinical observation is a treatment-emergent adverse event, the event should be reported.

Very Likely/Certain:	A clinical event with a plausible time relationship to vaccine administration and which cannot be explained by concurrent disease or other drugs or chemicals.
Probable:	A clinical event with a reasonable time relationship to vaccine administration; is unlikely to be attributed to concurrent disease or other drugs or chemicals.
Possible:	A clinical event with a reasonable time relationship to vaccine administration, but which could also be explained by concurrent disease or other drugs or chemicals.
Unlikely:	A clinical event whose time relationship to vaccine administration makes a causal connection improbable, but which could be plausibly explained by underlying disease or other drugs or chemicals
Unrelated:	A clinical event with an incompatible time relationship and which could be explained by underlying disease or other drugs or chemicals.
Unclassifiable:	A clinical event with insufficient information to permit assessment and identification of the cause.

7.3 Assessment of Severity

The intensity of the adverse event will be rated adapting the guidelines, where applicable, set by the U.S. FDA Toxicity Grading Scale for Healthy Adult and Adolescent Volunteers Enrolled in Preventive Vaccine Clinical Trials[17] except for mild diarrhea, which will be defined as 3 loose or liquid stools in a 24 hour period.

**Systemic (General)**	**Mild (Grade 1)**	**Moderate (Grade 2)**	**Severe (Grade 3)**	**Potentially Life Threatening (Grade 4)**
Nausea/vomiting	No interference with activity or 1 - 2 episodes/24 hours	Some interference with activity or > 2 episodes/24 hours	Prevents daily activity, requires outpatient IV hydration	ER visit or hospitalization for hypotensive shock
Diarrhea	3 loose stools or < 400 gms/24 hours	4 - 5 stools or 400 - 800 gms/24 hours	6 or more watery stools or > 800gms/24 hours or requires outpatient IV hydration	ER visit or hospitalization
Headache	No interference with activity	Repeated use of non-narcotic pain reliever > 24 hours or some interference with activity	Significant; any use of narcotic pain reliever or prevents daily activity	ER visit or hospitalization
Fatigue	No interference with activity	Some interference with activity	Significant; prevents daily activity	ER visit or hospitalization

**Systemic Illness**	**Mild (Grade 1)**	**Moderate (Grade 2)**	**Severe (Grade 3)**	**Potentially Life Threatening (Grade 4)**
Illness or clinical adverse event (as defined according to applicable regulations)	No interference with activity	Some interference with activity not requiring medical intervention	Prevents daily activity and requires medical intervention	ER visit or hospitalization

Changes in the severity of an adverse event should be documented to allow an assessment of the duration of the event at each level of intensity to be performed. Adverse events characterized as intermittent require documentation of onset and duration of each episode.

7.4 Reporting of Serious Adverse Events

Serious adverse events will be reported promptly once the principal investigator or designee determines that the event meets the protocol definition of a SAE. The principal investigator or designee will provide an assessment of causality.

- The investigator or designee will fax or email the SAE report to the DSMB and to IVI (through the monitor), and to Shantha Biotechnics (through the medical monitor, Dr Mandeep Singh Dhingra) within 24 hours of his/her becoming aware of these events.

- The SAE form will always be completed as thoroughly as possible with all available details of the event, assessment of causality, and signed by the investigator (or designee). If the investigator does not have all information regarding an SAE, he/she will not wait to receive additional information before notifying the DSMB, IVI, and Shantha Biotechnics.

- After the initial SAE report, the investigator is required to proactively follow each subject and provide further information to the DSMB, IVI, and Shantha Biotechnics on the subject's condition. The investigator (or designee) will follow-up subjects with SAEs until the event has: resolved, subsided, stabilized, or disappeared or the event is otherwise explained, or the subject is lost to follow-up.

- The date of final disappearance of the adverse event will be documented.

- The principal investigator (or designee) will always provide an assessment of causality at the time of the initial report. The DSMB may request that the investigator perform or arrange for the conduct of supplemental measurements and/or evaluations to elucidate as fully as possible the nature and/or causality of the serious adverse event. The investigator is obliged to assist. If deemed necessary by one or more SAEs, the DSMB will have the authority to call a temporary moratorium on vaccination, so that the DSMB can review the adverse events data.

8 DATA COLLECTION AND MANAGEMENT PROCEDURES

8.1 Case Report Forms (CRF)

Case report forms (CRF) and diary cards (if applicable) will be maintained for recording data for each subject enrolled in the study. The investigator is responsible to ensure the accuracy, completeness, legibility and timeliness of the data reported to the sponsor in the CRFs, spreadsheets and diary cards. Data reported in the CRFs and spreadsheets derived from source documents should be consistent with the source documents or the discrepancies should be explained. The IVI and Shantha will provide guidance to investigators on making corrections to the CRF and spreadsheets.

8.2 Source Documentation

Each subject requires complete and adequate source documentation (hospital or medical records, lab reports, test results) for the complete period of the study, unless the data recorded directly on the

case report form is considered the source data. These records must be available to the IVI and regulatory authorities upon request for review.

8.3 Data Management

Study personnel will extract all data collected in CRFs for computerization. Data will be double-entered into computers using a data entry program specially created for the project by the IVI.

These programs will utilize custom-made software; all programs will incorporate identification of the keypunching errors, range and consistency checks *pari passu* with data entry. This software will provide error reports, exception lists, and summary reports for each activity. The software will also automatically back-up data at systematic intervals onto local hard disks and external medias, and will provide for an audit trail of all sequential changes made.

Data security for this data management system will be maintained with password protection for accessing the data and data management software. In addition, backup files generated by the data management software will be kept in a secure cabinet.

Data entry and cleaning will be conducted at Shantha Biotechnics and the IVI will be the data coordinating center. Final data cleaning, data freezing and data analysis will be performed at IVI.

9 STATISTICAL CONSIDERATIONS

9.1 Sample Size Calculation

For diarrheal adverse events in the study, we assume 1) the background rate of diarrhea in the placebo group will be 5% after each dose; 2) the true rate of diarrhea is the same in the vaccine and placebo groups; 3) we wish to exclude, for the vaccine, a one-tailed 95% CI for the vaccine-placebo difference in the rate of diarrhea greater than 20%, with .8 power. With these assumptions, and using the method of Farrington and Manning for precision-based sample size calculations[18], a total of 31 subjects per group would be needed.

For serum vibriocidal responses (defined as ≥ 4-fold increases between baseline and post-second dose) in the study, we assume 1) the background rate of responses in the placebo group will be 5% after the second dose; 2) the true rate of vibriocidal responses in the vaccine group is 60%; 3) we wish to exclude, for the vaccine, a one-tailed lower 95% CI for the vaccine-placebo difference of lower than 30% with .9 power. With these assumptions, and using the method of Farrington and Manning for precision-based sample size calculations[18], a total of 41 subjects per group would be needed.

If we assume a dropout rate between first dose and the second bleed will be 20%, a total of 100 adult subjects and 100 children subjects will be needed.

9.2 Analysis Plans

Both intention-to-vaccinate and per protocol analysis will be performed.

Intention-to-Vaccinate Analysis

Every subject randomized in the study will be analyzed, except if he/she did not receive any dose of the study agent or if no data was collected for this subject.

Per-Protocol Analysis

A per protocol analysis will compare subjects according to the study agent actually received and will include only those subjects who satisfied the inclusion/exclusion criteria, followed the protocol, completed all visits and received the correct dose. The following non-compliant subjects will be excluded:

- Subjects included without meeting at least one inclusion criterion
- Subjects included despite meeting at least one exclusion criterion
- Subjects found non compliant with the blood sampling schedule.
- Subjects excluded from the intention to vaccinate analysis.

Analysis of demographics

Demographic characteristics of subjects enrolled will be tabulated.

Analysis of safety

Any adverse event that occurs prior to vaccination will not be included in safety analysis. The number and percentage of subjects (with 95% CI) with diarrheal adverse event will be compared. In addition, the number and percentage of subjects (with 95% CI) with at least one adverse event (solicited and/or unsolicited) after vaccination and during the 4 weeks follow up period will be compared between the study groups.

The number and percentage of subjects with at least one Serious Adverse Event, with the frequencies of each type of event will be compared between the study groups.

Over-all rates of adverse reaction will be analyzed using the chi-square test or by the Fisher's exact test when the numbers are sparse.

Analysis of immunogenicity (vibriocidal immune response)

Demonstration of a fourfold or greater rise in serum anti-O1 vibriocidal antibody titer will be the primary measure of vaccine immunogenicity. Geometric mean fold rises of serum titres will also be analyzed and compared. The number and percentage of subjects (with 95% CI) who exhibit at least a fourfold rise in serum anti-O1 vibriocidal titer after vaccination will be compared with the historical data available from the phase II and Phase III studies with the vaccine.[14-16]

Serum vibriocidal titers and fold-rises may be logarithmically transformed prior to statistical analyses in order to better approximate normality. Student's t-test will be performed for

continuous outcomes. Seroconversion will be compared using the chi-square test with Yates correction or by the Fisher's exact test if the numbers are sparse. Analysis of covariance may be used to adjust for imbalances in baseline titers.

Interim analysis

No interim analysis is planned.

10 MONITORING, AUDITING, INSPECTION

10.1 Responsibilities of the investigator(s)

The site principal investigator will conduct the study in accordance with this protocol and will attempt to recruit the required number of patients in a reasonable period of time so as to complete the trial at the earliest. The site principal investigator will provide copies of the protocol to all the members of his study team. He or she will discuss this material with them and conduct training to assure that all the members of their study team are fully informed regarding the vaccine/placebo and the conduct of the study. He or she will ensure that all his associates, colleagues and employees assisting in the conduct of this study are informed about their obligations in meeting their respective commitments. The principal investigator will provide a final report of the study.

The site principal investigator will ensure that all case report forms will be completed and computerized in real time (that is within 24 to 48 hours of completion of the form) to assure accurate and timely data. Any forms with queries or inconsistencies noted during data entry will be sent back to the study clinic for correction or clarification.

10.2 Responsibilities of the IVI coordinators and Shantha Monitors

The coordinators from IVI and monitors from Shantha Biotechnics Limited will ensure that the trial is adequately monitored. At regular intervals, contact with the study site will be made through visits, e-mail, and telephone calls to review the study progress, adherence to the protocol, and any problems. During the monitoring visits, the following will be examined: subject informed consent, subject recruitment and follow-up, vaccine allocation, vaccine storage and transport, follow-up of subjects, and laboratory procedures. The coordinators and monitors will discuss any problems with the investigators and define, after deliberation, any action(s) to be taken.

10.3 Responsibilities of the DSMB

A data safety monitoring board (DSMB) will be established to assess at intervals the progress of the trial and the safety of the study agent. This will be constituted by individuals whose field of expertise includes cholera, diarrhea and infectious diseases. The DSMB will be independent of and separate from the activities of the IVI staff. They will evaluate safety information, including SAEs, at mid-point of recruitment, and again after all subjects have completed dosing. More frequent and/or ad hoc meetings may be convened at the request of the DSMB and/or sponsor.

11 ETHICAL AND REGULATORY STANDARDS

11.1 Ethical principles/ Laws and regulations

The study will be conducted in compliance with the procedures outlined in this protocol, the International Conference on Harmonization's Good Clinical Practice Guidelines (ICH-GCP) and in accordance with the ethical guidelines and local regulatory requirements for the trial. It is also expected that local ethics committees will follow guidelines set forth by WHO to ensure quality of the ethical review.

11.2 Potential risks and risk minimization

Risk-benefit. The killed bivalent whole cell oral cholera vaccine has not been reported to be associated with major adverse reactions in the course of the phase III trial performed in Kolkata. The potential benefits to participants are substantial, since cholera is endemic in many parts of India and the eventual licensure of this Indian produced vaccine will translate into accessibility of this vaccine in India and endemic countries worldwide.

Benefit to all participants. Knowledge regarding the use of this vaccine in a population with endemic cholera will be useful for future use of this vaccine in India. No pro-rated payment will be given, but reimbursement for transportation expenses and time lost from work will be given. Vitamins or food will be offered after each blood sample is taken.

11.3 Informed consent and Assent

In obtaining and documenting informed consent and assent, the investigator must comply with the applicable regulatory requirements, GCP guidelines and ethical principles. The written informed consent form and assent form must be approved by and Institutional Review Board/Ethics Committee (IRB/EC) prior to its use.

The written informed consent will be obtained by the study physicians prior to enrolment for all participants. Subjects, or their parents and/or guardians (if subject is less than 18 years of age) will read the informed consent, and be allowed to ask questions regarding the study. If the subject or parents/guardians can not read, the informed consent will be read and explained to them. The subject or their parent/guardian must sign (or a thumbprint will be placed, if illiterate) and date the informed consent form prior to participating in any study-related activity. A witness must sign the informed consent form if the subject or parent/guardian is illiterate. The informed consent form must be signed and dated by the study personnel who obtained the consent.

A written assent form will be obtained prior to enrolment for all participants aged 12 – 17 years. They will read the assent form, and be allowed to ask questions regarding the study. If the subject can not read, the assent will be read and explained to them. The subject will sign (or a thumbprint will be placed, if illiterate) and date the form prior to any study-related activity. A witness must sign the assent form if the subject is illiterate. In addition, the assent form must be signed and dated by the study personnel who obtained the assent.

If information becomes available that may be relevant to the subject's willingness to continue participating in the study, the investigator will inform the study subjects in a timely manner and a revised written informed consent must be obtained.

11.4 Institutional Review Committee (IRB) / Ethics Committee (EC)

Before initiation of the study, the final protocol, and appropriate documents (information to be given to the subjects, informed consent forms, subject recruitment procedures, if any, investigator's brochure, information sheets and advertisements) will be submitted to the IRB/EC of CMC, Vellore, and IVI by the investigators. A copy of the study approval (including the informed consent approval) is to be kept in the Investigator's study document binder and a copy is to be supplied to the IVI. Clearances from the appropriate local ethical review boards and the IVI Institutional Review Board will be obtained.

During the study, the investigator is responsible for providing the IRB/EC with all the documents subject to review (i.e. Protocol amendments, Informed consent updates, advertisements, and other written information to be provided to the subject). Appropriate reports on the progress and termination of the study will be made to the IRB/EC by the investigator in accordance with the IRB/EC guidelines and government regulations (if applicable)

Clearance to use the vaccine in a clinical trial will be obtained from the Drugs Controller General of India (DCGI). Clearance would also be obtained from DCGI to export the serum samples to IVI Korea for analysis.

12 ADMINISTRATIVE ASPECTS

12.1 Record retention

The investigator will retain trial related documents as required by the applicable regulatory requirement (s) or by an agreement with the sponsor. The investigator should take measure to prevent accidental or premature destruction of these documents.

Essential documents should be retained for:

- A period of two years after approval of a marketing application in an ICH region and until there is no pending or contemplated marketing applications in an ICH region.

  OR

- A period of two years has elapsed since the formal discontinuation of clinical development of the investigational product.

The essential documents could be retained for a longer period however, if required by applicable regulatory requirements or by an agreement with the sponsor. It is the responsibility of the IVI and

Shantha Biotechnics to inform the investigator as to when these documents no longer need to be retained.

12.2 Publications

IVI, Shantha Biotechnics and CMC, Vellore shall jointly own the rights to the data, clinical, and biological specimens, results and other findings resulting from this trial. The study results will be reported to the Drugs Controller General of India (DCGI). Parties are encouraged to publish the results of their work in a collaborative fashion for the benefit of the public while taking care to protect the intellectual property rights to proprietary discoveries. There shall be joint access of data. Guidelines for authorship of major, international, peer-reviewed journals will be used to establish authorship. Each party shall provide the others with a copy of each manuscript and abstract at least 30 days before submission for publication in a journal or presentation at an international meeting. The parties will have the right to examine the publication before it is printed and disseminated, and to request changes to the use of their name.

Subject to agreement on a case-by-case basis, each party is encouraged to produce and disseminate electronic versions of important publications produced as a result of this Cooperative Agreement. Each party will permit the others to disseminate such electronic versions as long as all original formatting, credits, and contents are maintained.

The contribution of all parties involved in this Cooperative Agreement shall be acknowledged in all abstracts, reports, or other peer-reviewed scientific publications containing data or information collected during the Project duration.

12.3 Study Funding

This trial is part of the CHOVI Programme funded by the Bill and Melinda Gates Foundation and coordinated by the International Vaccine Institute.

13. REFERENCES

1. World Health Organization. Cholera, 2006. Wkly Epidemiol Rec, 2007; 82(31): p. 273-284.

2. World Health Organization. State of the art of new vaccines:research and development. Revised 2005, Geneva, Switzerland. WHO/IVB/05.

3. Zuckerman, JN, Rombo L, Fisch A. The true burden and risk of cholera: implications for prevention and control. *Lancet* 2007; 7:521-30

4. Government of India. Health Information of India 1995, DGHS, New Delhi

5. Sur D, Dutta P, Nair GB, Bhattacharya SK. Severe cholera outbreak following floods in a northern district of West Bengal. *Indian J Med Res* 2000; 112: 178-82.

6. Radhakutty G, Sircar BK, Mondal SK, Mukhopadhyay AK, Mitra RK, Basu A, Ichpugani I, Nair GB, Bhattacharya SK. Investigation of an outbreak of cholera in Allepey and Palghat districts, South India. *Indian J Med Res* 1997; 106: 455-7.

7. Niyogi SK, Mondal S, Sarkar BL, Garg S, Banerjee D, Dey GN. Outbreak of cholera due to *V cholerae* O1 in Orissa state. *Indian J Med Res* 1994; 100: 217-8.

8. Ramarmurthy P, Garg S, Sharma R, Bhattacharya SK, Nair GB, Shimada T,, Karasawa T, Kurazono H, Pal A, Takeda Y. Emergence of a novel strain of *Vibrio cholerae* with epidemic potential in southern and eastern India. *The Lancet* 1993; 341: 703-4.

9. World Health Organization Global Task Force on Cholera Control. Cholera Vaccines: A new public health tool? Report of a WHO meeting, 10-11 December 2002, Geneva, Switzerland. WHO/CDS/CPE/ZFK/2004.5.

10. Clemens J D et al , Field trials of cholera vaccines in Bangladesh: results from a three year follow- up. *The Lancet* 1990; 335: 270 –3.

11. Sanchez J L, Vasquez B, Beque R et al, Protective efficacy of the oral whole cell / recombinant B subunit cholera vaccine in Peruvian military recruits. *The Lancet* 1994; 344: 1273 – 6.

12. Trach DD, Clemens JD, Ke NT, Thuy HT, Son ND, Canh DG, Hang PV, Rao MR. Field trial of a locally produced, killed,oral cholera vaccine in Vietnam. *The Lancet* 1997; 349, 231-5.

13. Trach DD, Cam PD, Ke NT, Rao MR, Dinh D, Hang PV, Hung NV, Canh DG, Thiem VD, Naficy A, Ivanoff B, Svennerholm AM, Holmgren J, Clemens JD. Investigations into the safety and immunogenicity of a killed oral cholera vaccine developed in Viet Nam. *Bulletin of the World Health Organization* 2002; 8, 2-8.

14. Anh DD, Lopez AL, Canh DG, Thiem VD, Sonh NN, et al. Safety and Immunogenicity of the reformulated Vietnamese bivalent killed, whole cell oral cholera vaccine in adults. *Vaccine* 2007; 25: 1149-1155.

15. Mahalanabis D, Lopez AL, Sur D, Deen J, Manna B, et.al. A randomized, placebo-controlled trial of the bivalent killed, whole-cell, oral cholera vaccine in adults and children in a cholera endemic area in Kolkata, India. *PLoS ONE* 2008: 3 (6): e2323. doi:10.1371/journal.pone.0002323.
16. Sur D, Lopez AL, Kanungo S, Paisley A, Manna B, et al. Efficacy and safety of a modified killed-whole-cell oral cholera vaccine in India: an interim analysis of a cluster-randomised, double-blind, placebo-controlled trial. Lancet 2009;374:1694-1702.

17. United States Food and Drugs Administration Center for Biologics Evaluation and Research. Toxicity Grading Scale for Healthy Adult and Adolescent Volunteers Enrolled in Preventive Vaccine Clinical Trials. available at http://www.fda.gov/cber/gdlns/toxvac.htm, accessed on 4 December 2007.

18. Farrington , Manning. Test statistics and sample size formulae for comparative binomial trials with null hypothesis of non-zero risk difference or non-unity relative risk. Stat Med 1990; 9:1447-54.
